# Detection and therapy of neuroblastoma minimal residual disease using [^64/67^Cu]Cu-SARTATE in a preclinical model of hepatic metastases

**DOI:** 10.1186/s13550-021-00763-0

**Published:** 2021-02-25

**Authors:** Jason L. J. Dearling, Ellen M. van Dam, Matthew J. Harris, Alan B. Packard

**Affiliations:** 1 Division of Nuclear Medicine and Molecular Imaging, Department of Radiology, Boston Children’s Hospital, 300 Longwood Ave, Boston, MA 02115 USA; 2 Harvard Medical School, Boston, MA 02115 USA; 3 Clarity Pharmaceuticals Ltd., 4 Cornwallis St., Sydney, NSW 2015 Australia

**Keywords:** PRRT, SARTATE, SSTR2, Neuroblastoma, MRD

## Abstract

**Background:**

A major challenge to the long-term success of neuroblastoma therapy is widespread metastases that survive initial therapy as minimal residual disease (MRD). The SSTR2 receptor is expressed by most neuroblastoma tumors making it an attractive target for molecularly targeted radionuclide therapy. SARTATE consists of octreotate, which targets the SSTR2 receptor, conjugated to MeCOSar, a bifunctional chelator with high affinity for copper. Cu-SARTATE offers the potential to both detect and treat neuroblastoma MRD by using [^64^Cu]Cu-SARTATE to detect and monitor the disease and [^67^Cu]Cu-SARTATE as the companion therapeutic agent. In the present study, we tested this theranostic pair in a preclinical model of neuroblastoma MRD. An intrahepatic model of metastatic neuroblastoma was established using IMR32 cells in nude mice. The biodistribution of [^64^Cu]Cu-SARTATE was measured using small-animal PET and ex vivo tissue analysis. Survival studies were carried out using the same model: mice (6–8 mice/group) were given single doses of saline, or 9.25 MBq (250 µCi), or 18.5 MBq (500 µCi) of [^67^Cu]Cu-SARTATE at either 2 or 4 weeks after tumor cell inoculation.

**Results:**

PET imaging and ex vivo biodistribution confirmed tumor uptake of [^64^Cu]Cu-SARTATE and rapid clearance from other tissues. The major clearance tissues were the kidneys (15.6 ± 5.8% IA/g at 24 h post-injection, 11.5 ± 2.8% IA/g at 48 h, *n* = 3/4). Autoradiography and histological analysis confirmed [^64^Cu]Cu-SARTATE uptake in viable, SSTR2-positive tumor regions with mean tumor uptakes of 14.1–25.0% IA/g at 24 h. [^67^Cu]Cu-SARTATE therapy was effective when started 2 weeks after tumor cell inoculation, extending survival by an average of 13 days (30%) compared with the untreated group (mean survival of control group 43.0 ± 8.1 days vs. 55.6 ± 9.1 days for the treated group; *p* = 0.012). No significant therapeutic effect was observed when [^67^Cu]Cu-SARTATE was started 4 weeks after tumor cell inoculation, when the tumors would have been larger (control group 14.6 ± 8.5 days; 9.25 MBq group 9.5 ± 1.6 days; 18.5 MBq group 15.6 ± 4.1 days; *p* = 0.064).

**Conclusions:**

Clinical experiences of peptide-receptor radionuclide therapy for metastatic disease have been encouraging. This study demonstrates the potential for a theranostic approach using [^64/67^Cu]Cu-SARTATE for the detection and treatment of SSTR2-positive neuroblastoma MRD.

## Background

Neuroblastoma (NB) is an early childhood cancer that accounts for approximately 15% of pediatric cancer deaths [1]. It presents as a highly heterogeneous disease in terms of clinical progression, pathology and therapy response [2]. In high-risk disease (*i.e.*, Stage III and IV), which comprises more than half of all cases, 5-year survival is typically less than 40% [3]. In these patients, metastases typically arise throughout the body post-therapy due to minimal residual disease (MRD), small deposits of tumor cells that evade initial therapy and subsequently grow to devastating effect [4].

The initial diagnosis of NB is typically by physical examination, measurement of catecholamines in urine and/or blood samples, and imaging including SPECT imaging using ^123^I- or ^131^I-labeled *meta*-iodobenzylguanidine (*m*IBG), a norepinephrine analog that is taken up by most neuroblastoma cells [5]. SPECT imaging using *m*IBG may be complemented by 2-[^18^F]FDG PET imaging [6]. However, there remains a need for an improved imaging agent for NB, preferably a PET imaging agent, that can be used both to evaluate the extent of disease and monitor response to therapy [6, 7].

Somatostatin receptor 2 (SSTR2) is expressed by many tumor types including the majority of NB tumors, with expression in clinical samples ranging from 77 to 100% depending on the method of detection [8–12], and there is a negative correlation between SSTR2 expression and clinical risk [10]. SSTR2 is, therefore, considered to be an important target for the development of somatostatin analogues for molecular imaging and radionuclide therapy [13]. In one example, the somatostatin analog octreotate was conjugated with the bifunctional chelator DOTA to generate DOTA-TATE and labeled with either ^68^Ga (NETSPOT^®^) for the localization of neuroendocrine tumors (NETs), or ^177^Lu (Lutathera^®^) for the treatment of gastroenteropancreatic (GEP) NETs [14–16]. A similar approach may be beneficial for the imaging and treatment of SSTR2-positive NB [17–19], and [^68^Ga]Ga-DOTA-TATE for PET imaging of SSTR2-positive neuroblastoma followed by treatment with either ^177^Lu- or ^90^Y-labeled DOTA-TATE has shown promising initial results [18, 20].

In contrast to ^68^Ga and ^177^Lu, ^64^Cu and ^67^Cu provide the ability to use a “true” theranostic pair of radionuclides for imaging and therapy: The biological behavior of the [^64^Cu]Cu-SARTATE imaging agent will be identical to that of the [^67^Cu]Cu-SARTATE therapeutic agent, which is not true of the ^68^Ga/^177^Lu pair. Copper-64 has a longer half-life than ^68^Ga (12.7 h vs. 68 min), which allows for imaging at longer times post-injection. This not only improves image contrast [21, 22], but also facilitates the calculation of patient-specific dosimetry. Furthermore, resolution of ^64^Cu PET images is superior to that of ^68^Ga due to its lower β^+^ energy (278 keV vs. 830 keV) [23]. The longer half-life of ^64^Cu also provides two significant practical advantages over ^68^Ga. First, it allows for centralized production and distribution of ^64^Cu-labeled radiopharmaceuticals to sites that may not have access to a ^68^Ge/^68^Ga generator, similar to the way that ^18^F-labeled radiopharmaceuticals are distributed to facilities without on-site cyclotrons. Second, there is minimal decay of the ^64^Cu if a significant amount of time is needed to position younger pediatric patients, who make up a large percentage of patients with  NB, prior to either injection or scanning. With respect to therapy, the half-life of ^67^Cu is 62 h compared to 6.6 d for ^177^Lu, and the β^−^ yields (both 100%) and energies (^67^Cu 141 keV, ^177^Lu 134 keV) are very similar. The combination of ^64^Cu and ^67^Cu therefore offers several advantages over ^68^Ga/^177^Lu as a theranostic pair.

**Fig. 1:**
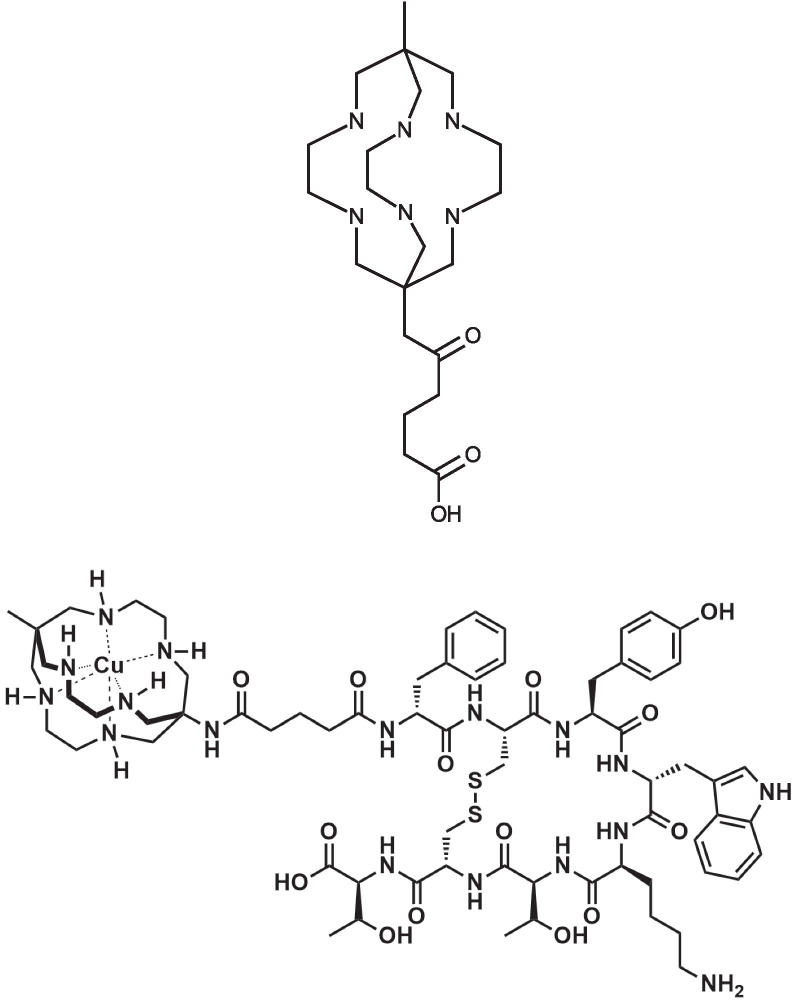
Chemical structures. The chemical structures of MeCOSar (top) and Cu-SARTATE (bottom) are shown

While DOTA forms complexes with ^68^Ga and ^177^Lu that are stable in vivo, the DOTA complex with ^64^Cu is not stable in vivo [24]. To address this limitation, Paterson et al*.* [25] developed SARTATE, an SSTR2-targeted octreotate conjugate that incorporates the novel sarcophagine chelator, MeCOSar (Fig. 1). MeCOSar, derived from SarAr [26], forms complexes with copper radionuclides that are extremely stable in vivo. In a first-in-human PET/CT imaging study, [^64^Cu]Cu-SARTATE compared favorably to [^68^Ga]Ga-DOTATATE, especially for liver lesions, in patients with neuroendocrine tumors [27]. The therapeutic potential of [^67^Cu]Cu-SARTATE was recently demonstrated in a mouse model of rat pancreatic exocrine tumors in which it inhibited tumor growth and increased survival [28], and a clinical trial using this agent to treat neuroblastoma is currently underway [29].

In this study, the biodistribution of [^64^Cu]Cu-SARTATE was measured in an intrahepatic model of NB metastatic disease representing MRD, and the potential of [^67^Cu]Cu-SARTATE as a therapeutic agent for the treatment of metastatic NB was evaluated.

## Methods

### General

All chemicals and reagents were obtained from Sigma-Aldrich (St Louis, MO) unless otherwise stated. Cell culture reagents were obtained from Mediatech (Herndon, VA) unless otherwise stated. Glassware was washed with 2 M nitric acid and rinsed with ultrapure water (> 15 MΩ resistivity) (US Filter/Siemens Water Technologies, Warrendale, PA) before use. All solutions were prepared using ultrapure water. Metal contaminants were further reduced by passing solutions through a Chelex-100 column (Bio-Rad Laboratories, Hercules, CA). Metal-naive pipette tips were purchased from Rainin Instrument (Oakland, CA). Copper-64 was purchased from Washington University—St. Louis (St. Louis, MO) [30] and was supplied in 0.04 M HCl. For the biodistribution study, the specific activity of ^64^Cu was 2.05 GBq/µg (specific concentration: 41.7 MBq/µl); and for the imaging study, the specific activity was 3.94 GBq/µg (36.5 MBq/µl). All activities were corrected to the start of peptide radiolabeling. Copper-67 was purchased from the Idaho Accelerator Center (Idaho State University, Pocatello, ID) and was supplied in 0.05–0.1 M HCl. The specific activity of the ^67^Cu was 3.24 GBq/µg (specific concentration: 3.37 MBq/µl) (^67^Cu radionuclide purity (HPGe) > 99.7%) at the beginning of radiosynthesis for the first study and 8.76 GBq/µg (7.84 MBq/µl) (^67^Cu radionuclide purity (HPGe) > 99.9%) for the second. SARTATE (MW 1459 g/mol) was prepared by Auspep (Melbourne, Australia) using a modified version of a method reported previously [25]. Radioactivity in the tissue samples was assayed with a Packard Cobra II automated gamma counter (Meriden, CT). Human IMR32 neuroblastoma cells [31] were purchased from the American Type Culture Collection (Manassas, VA). Athymic (nu/nu) female mice (6–8 weeks of age, 20–25 g) were purchased from The Jackson Laboratory; Bar Harbor, ME.

Small-animal image data were obtained using a Bruker Albira multimodality (PET/SPECT/CT) imaging system (Bruker Corporation; Woodbridge CT). Phosphor storage screens were scanned using a Fujifilm BAS-5000 (FUJIFILM Life Science, Stamford CT).

### Radiolabeling

SARTATE was radiolabeled at room temperature for 15 min with either ^64^Cu or ^67^Cu using a modified version of a method previously described [25]. Typical labeling procedures are provided as examples.

For biodistribution studies, a vial containing lyophilized SARTATE was reconstituted with phosphate buffer (0.1 M, pH 7) to give a 1 mg/mL solution. A 2.5 µL aliquot of this solution (2.5 µg) was added to 16.5 µL of phosphate buffer (0.1 M, pH 5.0) containing 71.78 MBq (1.94 mCi) of ^64^Cu. The solution was mixed by vortexing, centrifuged briefly (2000 rpm for 10 s) to ensure that all of the materials were collected in the bottom of the tube and then incubated at room temperature for 15 min. Labeling yield was determined by thin-layer chromatography (TLC). A 1 µL aliquot of the reaction mixture was added to 10 µL of phosphate buffer (0.1 M, pH 7.0) containing ethylenediaminetetraacetic acid (EDTA, 10 mM). This solution was mixed, and 1 µL was spotted on a Si60 silica gel TLC strip, allowed to dry and then developed with 0.1 M phosphate buffer/10 mM EDTA solution. The TLC plates were cut in half, and radioactivity in the sections was assayed to measure ^64^Cu incorporation. The ^64^Cu-labeled SARTATE solution was diluted with saline and sterile filtered (0.2 µm) before injection.

For therapy studies, to 31 µL of 0.01 M HCl containing 243.1 MBq (6.57 mCi) of ^67^Cu was added 169 µL of phosphate buffer (0.1 M, pH 7). The lyophilized SARTATE vial was reconstituted as before and 4 µL (4 µg) of the solution was added to the ^67^Cu, mixed by vortexing, centrifuged briefly as before, and then incubated at room temperature for 15 min. The ^67^Cu-labeled SARTATE solution was diluted with saline and sterile filtered (0.2 µm) before injection.

### Metastatic model of neuroblastoma

A metastatic model of hepatic NB was established using the human IMR32 NB cells. Mice were anesthetized by isoflurane at 1–4% in a flow of oxygen, the spleen was exteriorized through an incision on the left side, and 1 × 10^6^ cells in 50 µl (PBS, 0.1 M, pH 7.4) were injected into the spleen, followed after 2 min by splenectomy. The inner wound was sutured and the outer wound was closed using metal clips which were removed approximately 9 days post-surgery. For analgesia, meloxicam (5 mg/kg, subcutaneous) was administered perioperatively for 48 h.

### Imaging and biodistribution studies

[^64^Cu]Cu-SARTATE was injected into the tail vein of the tumor-bearing mice (mean 3.61 MBq (97.7 µCi; mass of peptide injected per mouse = 0.126 µg) in 100 µL saline) and PET and CT data were collected at 1, 5, 24 and 48 h post-injection (p.i., *n* = 4–7). Mice were anesthetized with isoflurane at 1–4% in a flow of oxygen, and data were collected over 30 min. Blood was sampled by tail nick immediately after imaging. After the 24-h and 48-h imaging time points, mice (*n* = 3–4) were euthanized and selected tissues were excised, weighed and counted. Injectate standards were also counted for calculation of percentage of injected dose per gram of tissue (% IA/g). PET and CT images were registered manually using AMIDE software [32]. Data from volumes of interest (VOIs) were used to calculate the ^64^Cu concentration in selected tissues.

### Autoradiography and histology

Samples of liver containing metastases were taken for histological analysis. The tissues were frozen, sectioned (16 µm slice thickness), fixed in ice-cold methanol, air-dried and then exposed to phosphor storage plates. They were then stained using either hematoxylin and eosin to demonstrate general morphology or immunohistochemistry (IHC) to demonstrate SSTR2 expression (rabbit anti-SSTR2 polyclonal antibody: Novus Biologicals; Centennial, CO). Autoradiographs were analyzed using ImageJ. Regions of interest were drawn in areas corresponding to background, normal (i.e., non-tumor) liver, viable tumor and necrotic tumor, where available. At least, 10 sections of each tissue sample were analyzed. Injectate standards were prepared and exposed to phosphor storage plates so that counts per pixel from the tissue autoradiographs could be converted to % IA/g, according to the method described by Mies et al. [33].

### Molecularly targeted radiotherapy

Two therapy studies, differing in the tumor growth time before therapy was started, were carried out to measure the effect of [^67^Cu]Cu-SARTATE on the survival of mice with intrahepatic NB metastases.

In the first therapy study, the intrahepatic NB metastasis model was established in 20 mice as described above, and 3 weeks after tumor inoculation the animals were divided into three groups: control (*n* = 7), low dose (*n* = 6) and high dose (*n* = 7). Two animals from each group were injected with [^64^Cu]Cu-SARTATE and imaged using PET/CT at 24 h p.i. The mice were treated with [^67^Cu]Cu-SARTATE 1 week later (4 weeks after tumor inoculation). The control group was treated with saline, the low-dose group was treated with 9.25 MBq (250 µCi; 0.160 µg) [^67^Cu]Cu-SARTATE, and the high-dose group was treated with 18.5 MBq (500 µCi; 0.320 µg) of [^67^Cu]Cu-SARTATE, all injected via the tail vein. Two mice from each treated group were imaged at 24 h p.i. Data were collected over 30 min (60 s per projection) using a multipinhole collimator and a single field of view. The highest mass of peptide administered (0.32 µg) is > 15,000 × lower than the amount of octreotide required to elicit tumor growth control in previous studies [34, 35].

In the second therapy study, the intrahepatic NB metastasis model was established in 16 mice as described above, and 2 weeks after tumor inoculation the animals were treated with either saline (control, *n* = 8) or 18.5 MBq (500 µCi) of [^67^Cu]Cu-SARTATE (*n* = 8). Again, two mice from the treated group were imaged at 24 h p.i.

During therapy studies, the mice were monitored visually and weighed at least twice a week following tumor cell inoculation and splenectomy. They were removed from the study (by CO_2_ euthanasia) when one of the exclusion criteria (infection at the wound site, weight loss of ≥ 20%, distention of the abdomen by tumor growth and morbidity) was met.

### Statistical analysis

Data presented are means ± standard deviation, unless otherwise stated. Statistical differences were analyzed using Student’s *t* test, and *p* values were considered to be significant at the 5% level (*p* = 0.05). Survival data were analyzed using the Mantel-Cox log rank test. Statistical analysis was carried out using SPSS V 24 for Windows.

## Results

### Synthesis of [^64^Cu]Cu-SARTATE and [^67^Cu]Cu-SARTATE

[^64^Cu]Cu-SARTATE and [^67^Cu]Cu-SARTATE were prepared in phosphate buffer at room temperature in 15 min with a radiochemical yield of over 95% as assessed by radio-TLC. Under the conditions described, radiolabeled peptide remains on the baseline (Rf < 0.1) and uncomplexed radiocopper moves with the solvent front (Rf > 0.9). Radiochromatogram TLC analyses confirmed radiolabeling of > 95%.

### [^64^Cu]Cu-SARTATE is taken up by SSTR2 positive hepatic lesions in a metastatic model of NB

**Fig. 2:**
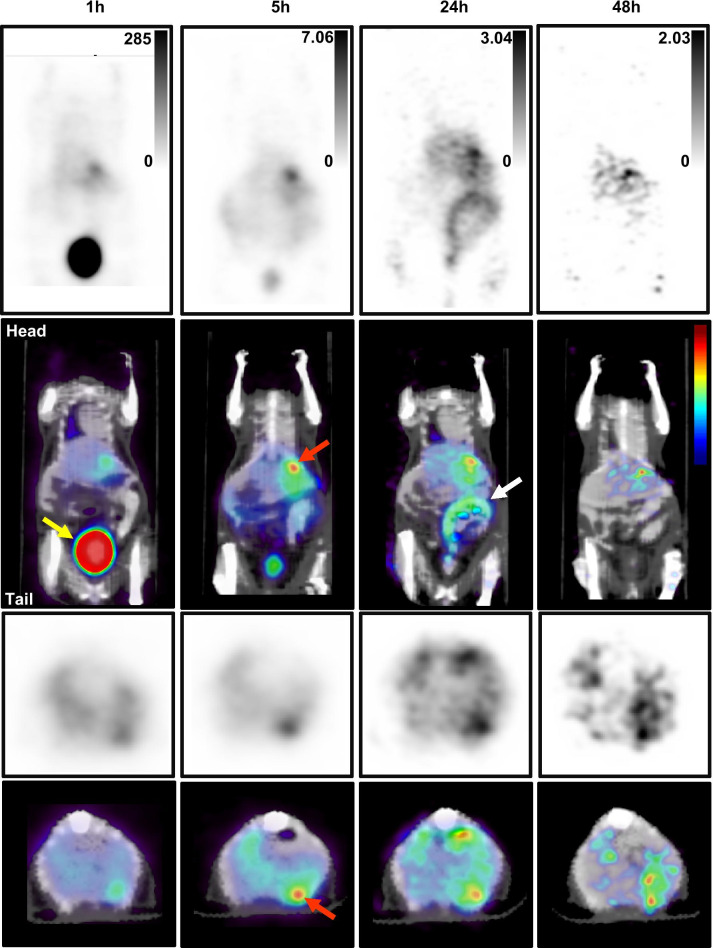
Small animal positron emission tomography images of a mouse following injection of ^64^Cu-SARTATE. PET/CT Image data were collected at 1, 5, 24 and 48 h post-injection. Mice were anesthetized with isoflurane at 1–4% in a flow of oxygen and data were collected over 30 min using a Bruker Albira PET/SPECT/CT camera. Representative coronal (top and second rows) and transaxial (third and bottom rows) images from one mouse are shown focusing on uptake in ventral intrahepatic metastases. Uptake in the  NB metastases (e.g., tumor indicated by red arrow in 5 h image) was evident throughout the study. The radiolabeled peptide cleared rapidly from most tissues with notable uptake in the kidneys, and liver/tumor. Radioactivity cleared via the bladder (yellow arrow, 1 h image). Clearance via the digestive tract (24 h, white arrow) was also detected. (Scale bars on the top row indicate maximum counts in the images in percentage of injected dose per gram (%IA/g), with no background subtraction.)

Expression of SSTR2 in IMR32 cells was confirmed by FACS analysis (Additional file 1: Fig. S1). On days 3–7 after tumor inoculation/splenectomy, the mice experienced a weight loss of approximately 8%, which was regained by days 14–16 after which they all gained weight at a similar rate (Additional file 1: Fig. S2). Extensive growth of intrahepatic metastases, confirmed at necropsy, was the removal criterion in all cases. Mice were administered ~ 3.5 MBq of [^64^Cu]Cu-SARTATE 4 weeks post-tumor inoculation/splenectomy. Examples of PET/CT images collected at 1, 5, 24 and 48 h p.i. (*n* = 4–7) are shown in Fig. 2. As expected, the highest tracer uptake was observed in the kidneys, as the primary clearance organ, followed by focal uptake in the liver. The heterogeneous [^64^Cu]Cu-SARTATE distribution in the liver was interpreted as uptake in dispersed tumor deposits.

**Fig. 3:**
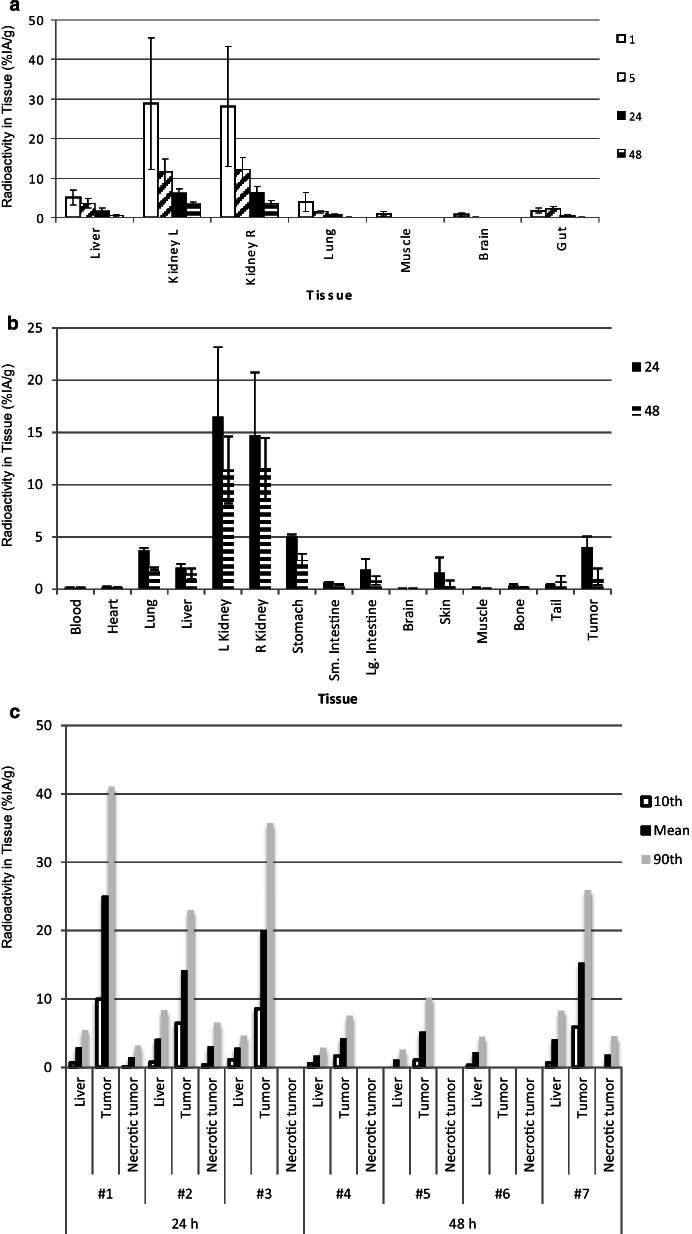
Biodistribution of ^64^Cu-SARTATE in mice-bearing IMR32 intrahepatic metastases. **a** PET images were analyzed using AMIDE (*n* = 4–7). **b** Data from ex vivo analysis of tissues at 24 h (*n* = 3) and 48 h (*n* = 4) post-injection. (Means ± SD). **c** Following exposure to phosphor storage plates and staining, regions of tissue samples were analyzed using ImageJ. Radionuclide uptake in liver, tumor and necrotic tumor are shown (10th percentile, mean and 90^th^ percentile data). No tumor was detected in sample #6, and no necrotic tumor was detected in samples #3 or 4

**Fig. 4:**
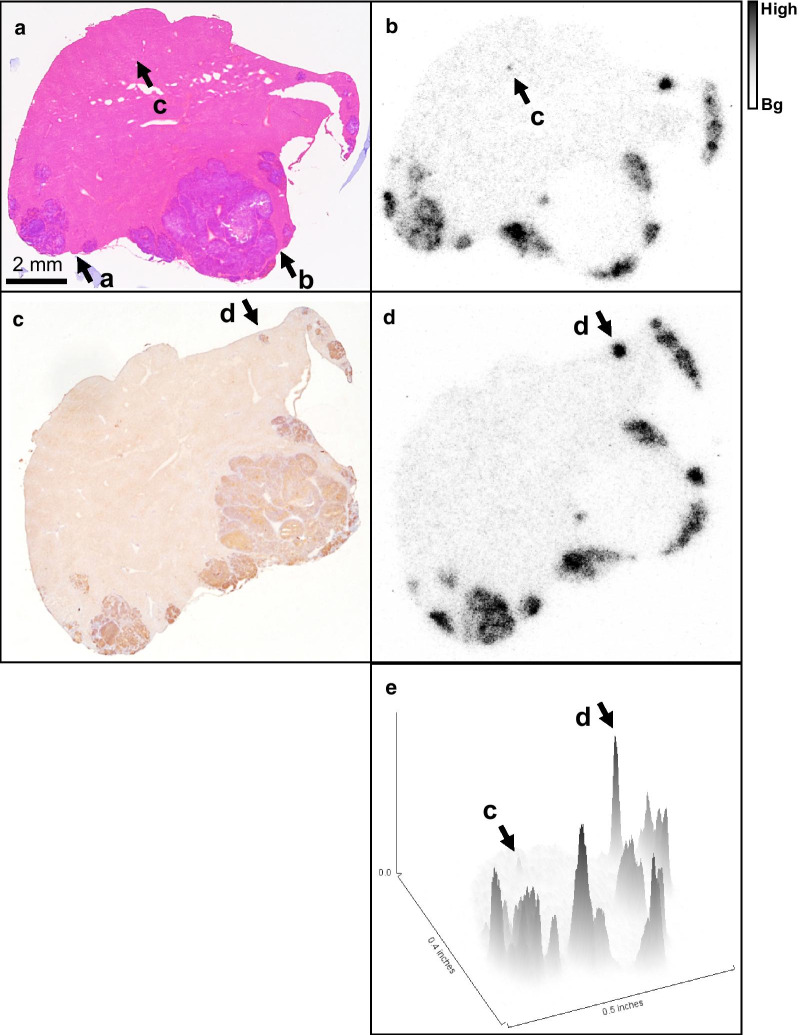
Images of tumor histology and autoradiography data showing distribution of ^64^Cu-SARTATE in tumor deposits and surrounding liver. Top row **a** shows a hematoxylin and eosin-stained frozen section of liver tissue (pink) containing darker purple tumor deposits and its corresponding autoradiograph (**b**) (size scale bar bottom left of **a**, autoradiography data scale bar right of **b**). Details of regions indicated by lower case a–d are shown in figure. Middle row **c** shows a contiguous section that has been stained using immunohistochemistry to demonstrate SSTR2 expression (dark brown) counter-stained with hematoxylin (blue) and its autoradiograph (**d**). Uptake of the radiolabeled peptide was highest in regions of tumor that were both viable and stained strongly for the target SSTR2. Bottom row (**e**) shows a surface plot of the autoradiograph B generated in ImageJ showing the relative uptakes in liver, viable SSTR2-positive tumor and necrotic tumor. Uptake in the small group of cells surrounded by liver “c” and the small SSTR2-positive deposit “d” are indicated with arrows for orientation

**Fig. 5:**
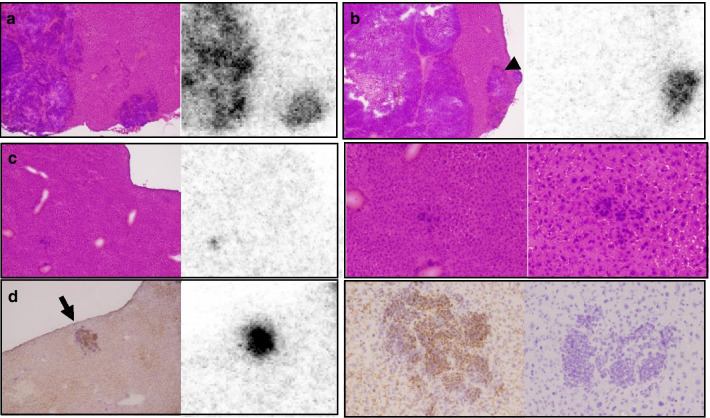
Detail from Fig. 4. Radionuclide distribution was heterogeneous in the viable tumor to the left of region **a**, and in the small deposit to the right with low uptake in the surrounding liver. There was high uptake in a small, viable deposit to the right of region **b** but very low uptake in the large, necrotic mass to the left. In region **c**, there was high uptake in an isolated group of cells (40 × magnification), and its corresponding autoradiograph. Images of the same region of cells (middle row, right and far right, photographed at 100 × and 200 × magnification) demonstrate the ability of ^64^Cu-SARTATE to target small tumor deposits. Region **d** refers to a tumor deposit that stained strongly for SSTR2 by IHC, then the corresponding autoradiograph showing high uptake in the tumor and low uptake in the surrounding liver. The next image is a × 200 magnification photograph of the same deposit and finally (right) the primary omission control of the same region from a contiguous tissue section confirming specificity of staining

The biodistribution of [^64^Cu]Cu-SARTATE is shown in Fig. 3. Clearance from the blood was rapid, decreasing from a mean of 3.6 ± 1.2% IA/g (mean ± ESD) at 1 h to 2.1 ± 1.9% IA/g at 5 h, then to 0.16 ± 0.06 and 0.1 ± 0.04% IA/g at 24 and 48 h, respectively (*n* = 4–7). [^64^Cu]Cu-SARTATE cleared rapidly from the kidneys, decreasing from 28.5 ± 15.9% IA/g (mean ± ESD) at 1 h p.i. to 3.4 ± 0.65% IA/g at 48 h. The tracer concentration in samples of tumor and liver was measured at the macroscopic level, but the nature of the model, with very small tumor deposits distributed throughout the liver, makes this especially challenging. This challenge was circumvented by using autoradiography to measure the intrahepatic distribution of [^64^Cu]Cu-SARTATE. Comparison of images of the same tissue sections stained to demonstrate either general morphology or SSTR2 expression revealed high [^64^Cu]Cu-SARTATE uptake in viable, SSTR2-positive NB metastases (Figs. 3c, 4, 5). Radionuclide uptake in liver, tumor and necrotic tumor samples from individual mice at 24 h and 48 h were converted to % IA/g using a standard curve (Additional file 1: Fig. S3) and are summarized in Fig. 3c, showing 10^th^ percentile, mean and 90^th^ percentile values to provide a measure of the range of values. While there was some degree of variation in absolute numbers, tumor uptake was consistently much higher than liver, with tumor-to-liver ratios of between 3.5 and 8.9 at 24 h, which decreased to 2.5 to 5.1 at 48 h. Uptake in viable regions of tumor, though heterogeneous, was always much higher than in necrotic regions. Figure 4a shows an example of an H&E-stained section, with liver staining pink and tumor staining purple. Comparison with the autoradiograph of the same section (Fig. 4b) indicates specific [^64^Cu]Cu-SARTATE uptake in the tumor. SSTR2 staining in the tumor is evident in the section below (Fig. 4c) and corresponds with high uptake in the autoradiogram of the same tissue sample (Fig. 4d). Figure 4e is a surface plot of radionuclide distribution in Fig. 4b, emphasizing the large difference in [^64^Cu]Cu-SARTATE uptake between the liver and the tumor. Figure 5 shows enlarged images from regions a to d of Fig. 4. Region a shows heterogenous uptake of [^64^Cu]Cu-SARTATE in a tumor. In region b, there is high uptake in the small deposit on the right, but very low uptake in the large, necrotic tumor. The absence of [^64^Cu]Cu-SARTATE may reflect decreased perfusion, which could be the cause of the tumor necrosis. Region c shows a very small group of tumor cells with high uptake of [^64^Cu]Cu-SARTATE surrounded by normal liver. The adjacent image shows the same regions at higher magnification. Finally, region d shows a deposit that stained strongly for SSTR2 and has high [^64^Cu]Cu-SARTATE uptake. A higher magnification image of this deposit and (far right) a high magnification image of the IHC primary omission control on a contiguous section clearly shows these are tumor cells expressing SSTR2. Mean uptake in this deposit (5d, arrow) was 51.7% IA/g. For comparison, mean uptake in a heterogeneous region of tumor uptake (tumor to the left of Fig. 5a) was 24.3% IA/g and mean uptake in the small tumor to the right of the necrotic region in region b (arrowhead, 5b) was 28.6% IA/g.

### Early treatment with [^67^Cu]Cu-SARTATE improves survival in a metastatic NB model

**Fig. 6:**
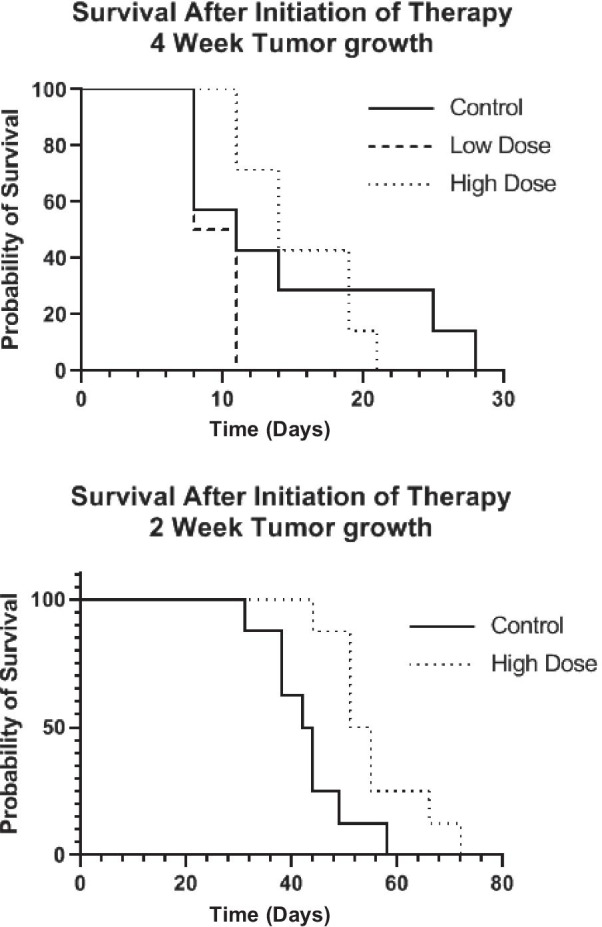
Analysis of survival data of mice in the two therapy studies. Kaplan–Meier curves describing the survival of mice-bearing intrahepatic neuroblastoma metastases and treated with ^67^Cu-SARTATE. The graphs show the survival of the two groups post-tumor cell inoculation and splenectomy. Mice in the first study (top) were treated 4 weeks after inoculation, and the second study (bottom) were treated 2 weeks after inoculation

**Fig. 7:**
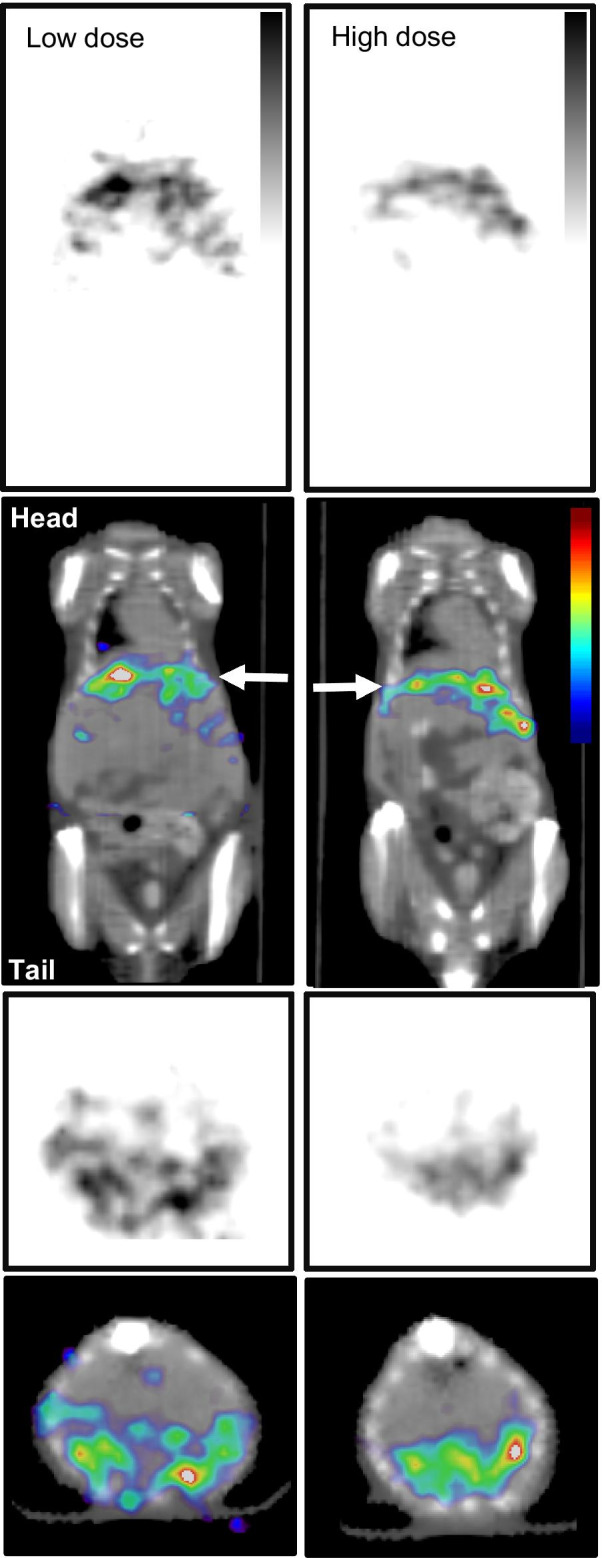
Single-photon emission computed tomography (SPECT) images of ^67^Cu-SARTATE distribution in mice-bearing intrahepatic NB metastases. Mice were injected with either 9.25 or 18.5 MBq (250 or 500 µCi) of radiolabeled peptide and imaged 24 h post-injection. Imaging anesthesia and instrumentation were as for the PET/CT imaging. A single field of view was used collimated by a multipinhole collimator, with data collected over 30 min (60 s per projection). Representative images of SPECT data in grayscale (top and third row) and SPECT data in color scale fused with CT data (second and bottom row) of mice injected with 9.14 MBq (247 µCi) (low dose, left column) or 18.9 MBq (510 µCi) (high dose, right column) of ^67^Cu-SARTATE are shown (*coronal*: top and second row,* transaxial*: third and bottom row). White arrows on the coronal images show the level of the transaxial slice. Color scale to right, CT for anatomic reference in gray scale

In order to determine the effect of tumor size on efficacy, radiotherapy with [^67^Cu]Cu-SARTATE was started either 2 weeks or 4 weeks after tumor inoculation/splenectomy. A peptide-only treatment control was omitted, as the amount of peptide mass given was > 15,000-fold less than that required to achieve tumor growth control, and we have not detected any difference between saline- and SARTATE-treated groups in previous studies. For these proof-of-concept studies, body weight was used as a surrogate for acute toxicity and all treatments were apparently well tolerated (Additional file 1: Fig. S2)*.* Kaplan–Meier survival graphs for both studies are presented in Fig. 6. For the cohort in which treatment began 4 weeks after tumor inoculation, [^64^Cu]Cu-SARTATE PET/CT imaging of selected mice from each treatment group was carried out in week 3 and confirmed SSTR2-positive NB tumors (Additional file 1: Fig. S5). Treatment starting in week 4 post-inoculation did not improve survival with mean (± SD) survival times of 14.6 ± 8.5 days (control), 9.5 ± 1.6 d (9.25 MBq) and 15.6 ± 4.1 (18.5 MBq) (survival analysis—log rank (Mantel-Cox) *p* = 0.064). Although the survival time of the 9.25 MBq group appears to be somewhat shorter than that of the untreated controls, the difference is not statistically significant. In the second study, [^67^Cu]Cu-SARTATE treatment was started 2 weeks after tumor cell inoculation, when the metastases were smaller. Representative SPECT/CT images are presented in Fig. 7, confirming uptake of the tracer in the tumors (red arrow). In this case, a significant extension of survival time of the [^67^Cu]Cu-SARTATE-treated group compared with untreated controls was observed: The control animals survived an average of 43.0 ± 8.1 d and the animals treated with 18.5 MBq [^67^Cu]Cu-SARTATE survived an average of 55.6 ± 9.1 d post-therapy (*p* = 0.012), a 30% improvement, even at this relatively low dose of ^67^Cu.

## Discussion

Paterson et al. [25] first reported superior tumor uptake and retention of [^64^Cu]Cu-SARTATE compared to [^64^Cu]Cu-DOTA-TATE in a subcutaneous lung cancer model overexpressing SSTR2 (A427-7). Further studies demonstrated the theranostic potential of [^64/67^Cu]Cu-SARTATE in a subcutaneous neuroendocrine model where [^67^Cu]Cu-SARTATE was shown to inhibit tumor growth and increase survival [28]. Targeting SSTR2 in NB was previously demonstrated clinically using ^68^Ga-, ^177^Lu- and ^90^Y-labeled DOTA-TATE [16, 18, 20], although a recent study failed to show a benefit of [^177^Lu]Lu-DOTA-TATE in NB patients, possibly due to the relatively low administered dose in this study [19].

NB patients often respond favorably to initial treatment, but subsequently relapse with extensive metastases, and these metastases most likely arise from MRD, small deposits of tumor cells that evade initial therapy [4]. We therefore aimed to investigate the potential of [^64/67^Cu]Cu-SARTATE as a theranostic pair to image and treat small-volume disease in an orthotopic model of metastatic NB. This model was selected because NB metastasis to the liver is a frequent clinical challenge, and in this model tumor growth is restricted to the liver, simplifying tissue sampling and monitoring during the therapy study. We measured the distribution of ^64^Cu-labeled SARTATE in a metastatic model of NB, demonstrated its localization in liver metastases using PET (^64^Cu) and SPECT (^67^Cu), and confirmed the uptake of [^64^Cu]Cu-SARTATE in viable, SSTR2-expressing regions of the liver metastases. We also demonstrated that ^67^Cu-labeled SARTATE significantly extended the lives of animals with NB liver metastases when treatment was initiated 2 weeks after tumor implantation.

One challenge of the model used in this study is that it is difficult to separate the small metastases from the normal liver. Clean dissection of the small metastases from normal liver is difficult and can result in a reduced apparent tracer uptake if the sample is contaminated by normal liver tissue. Conversely, apparently normal liver samples may contain small metastases with high tracer uptake, falsely increasing their apparent tracer uptake. In addition, quantification of tumor uptake in this model using small-animal PET imaging is challenging because the micrometastases are typically 1 mm in diameter or smaller, which is less than the camera resolution (~ 1.4 mm). These challenges were circumvented by using quantitative autoradiography, which confirmed the high uptake of [^64^Cu]Cu-SARTATE in the viable tumor and lower uptake in normal liver and necrotic tumor regions. These observations are in agreement with the preclinical study by Ullrich et al. [36] who reported the absence of expression of SSTR2 in necrotic regions of pheochromocytoma tumors. Also, Zhang et al. [37] reported the correlation of SSTR2 expression by CHLA-15 NB tumors with the localization of [^68^Ga]Ga-DOTA-TATE detected by autoradiography.

The specific, high and persistent tumor uptake of [^64^Cu]Cu-SARTATE provided a strong rationale for therapy studies with [^67^Cu]Cu-SARTATE. The first study, in which treatment was started at 4 weeks after tumor inoculation, showed no significant extension of survival in either of the treated groups compared with untreated controls. To better understand this result, a second study was carried out in which the mice were treated with [^67^Cu]Cu-SARTATE 2 weeks after tumor cell inoculation, when the tumors are smaller, and this study showed a significant extension in survival compared to untreated controls. An additional contributing factor may be that smaller tumors exhibit higher tracer uptake, as suggested by a previous study in NSCLC [38]. For the present study, however, no data are available relating tumor size to tracer uptake.

The results of this [^67^Cu]Cu-SARTATE therapy study in a NB model of hepatic metastases are in agreement with the results of previous preclinical PRRT studies in NB [36, 37], as well as other tumor models [28, 39, 40]. A single PRRT treatment did not lead to cure in any of these preclinical studies, but rather to some degree of tumor growth control and consequent survival extension. Either multiple PRRT treatments or a combination of PRRT with chemotherapy appears to be necessary in order to achieve a complete response [28, 39, 40]. It is important to note that these studies were carried out with relatively large subcutaneous tumors whereas we found that [^67^Cu]Cu-SARTATE therapy was significantly more successful in smaller, orthotopic, tumors.

As with any preclinical study, this study has its limitations. Our primary interest was in the verifying localization of the radiolabeled peptide in very small tumor deposits within the liver. Clinically, NB metastasizes to many tissues, but this model limits their location to the liver, simplifying sample analysis. Because the tumors are so widely disseminated in the liver, we were not able to quantify tumor growth in each mouse. This is not possible with PET imaging due to factors such as partial volume averaging and the spatial resolution of the camera. Other techniques such as bioluminescence imaging would have provided a measure of the total tumor cell population, but absolute quantitation is still challenged by other factors, such as tissue attenuation. In our initial therapy study, we began treatment when we knew that the tumors were established based on previous experience with this model (at 4 weeks post-tumor cell inoculation), and with a dose that was perhaps too low to see a significant difference between untreated and treated groups. We expected to achieve at least some tumor uptake, but we did not know if this dose would be high enough to achieve a therapeutic response, or if this dose would be toxic to other tissues, such as the kidney. Therefore, the doses we used were toward the lower end of those reported to result in tumor growth control in comparable studies. In the event, we did not encounter toxicity, which was only assessed by body weight, but did detect a 30% increase in survival time when mice were treated at 2 weeks post-inoculation. At this low dose, this was a very encouraging result in the context of a highly therapy resistant and metastatic pediatric cancer. Another limitation of this study is that a direct comparison with [^131^I]*m*IBG was not included in the treatment arm. However, a direct comparison of [^67^Cu]Cu-SARTATE and [^131^I]*m*IBG would be inherently complex, as it is known that expression of SSTR2 and the noradrenaline transporter (NAT) on neuroblastoma tumors does not overlap, which would introduce a confounding variable. The data in Fig. 6 appear to suggest that the control group in the first therapy study lived for significantly shorter than the controls in the second study (14.6 ± 8.5 days vs. 43 ± 8.1 days, respectively). This larger difference arises because the data are plotted post-start of therapy rather than post-inoculation of tumor cells, which gives a smaller difference between the two groups (45.6 ± 8.5 vs. 58 ± 8.1 days, respectively).

Minimal residual disease (MRD), millimeter-size deposits throughout the body that resist initial therapy, is a major challenge to durable NB therapy. High uptake of [^64^Cu]Cu-SARTATE in very small groups of tumor cells growing as orthotopic tumors was detected, confirming that [^64^Cu]Cu-SARTATE effectively targets these lesions. While [^131^I]*m*IBG remains a first-line treatment for NB, the use of [^67^Cu]Cu-SARTATE could complement [^131^I]*m*IBG therapy, reflecting the heterogeneity of tumor uptake by different lesions. Alternatively, it could serve as a follow-up to initial treatment as a way to treat MRD before it has a chance to grow into widespread metastases. This latter possibility is particularly intriguing because it suggests that a small dose of [^67^Cu]Cu-SARTATE given while NB patients are in remission might prevent the development of the metastases that very often appear months or years after initial treatment has been completed, increasing the durability of the therapy. Interestingly, in neuroendocrine tumor patients treated with [^177^Lu]Lu-DOTA-TATE [16], efficacy was not related to tumor burden [41], but instead the absence of a large target lesion (defined as > 3 cm) was associated with an improved progression-free survival (PFS). This result could be due to a tumor-sink effect, lower expression of SSTR2 on larger/necrotic tumors, or lower perfusion of larger tumors. Furthermore, it has been shown that medium-energy β^−^ emitters such as ^177^Lu and ^67^Cu are better suited for the irradiation of small, disseminated metastases, while the higher energy and longer range of the β^−^ particles emitted by ^90^Y make it more suitable for treating larger tumors [42, 43]. A practical advantage of ^67^Cu- and ^177^Lu-labeled therapeutic agents is that the lower-energy γ emissions of these radionuclides (^67^Cu, 184 keV [48.7%], 93 keV [16.1%]: ^177^Lu, 208 keV [10.4%], 113 keV [6.23%]: compared with ^131^I, 364 keV [81.5%], 637 keV [7.16%]) may eliminate the need for a dedicated shielded patient room which is sometimes required for high-dose [^131^I]*m*IBG therapy. Combining the dual benefits of reducing associated construction costs while also reducing the radiation dose to caregivers and the patients’ families increases the potential to use [^67^Cu]Cu-SARTATE to address the important clinical challenge of treating widespread metastases in recurrent neuroblastoma.

## Conclusion

[^64^Cu]Cu-SARTATE uptake was highest in the viable, SSTR2-positive regions of NB tumors, and cleared rapidly from non-tumor tissue. Treatment with [^67^Cu]Cu-SARTATE was more effective at 2 weeks post-tumor inoculation than at 4 weeks post-tumor inoculation in this model, suggesting that [^67^Cu]Cu-SARTATE could be more effective in smaller tumors that are more representative of minimal residual disease than in larger lesions. These results support the continued clinical evaluation of [^64^Cu]Cu-SARTATE for the detection and [^67^Cu]Cu-SARTATE for the treatment of SSTR2-positive NB.

## Supplementary Information

**Additional file 1**. Supplementary data. **Figure S1**. Fluorescence-activated cell sorting (FACS) analysis of IMR32 neuroblastoma cell SSTR2 expression. **Figure S2**. Body weights of groups of mice following splenectomy and during therapy. **Figure S3**. Standard curve for sensitivity of the phosphor storage plates. **Figure S4**. Immunohistochemistry positive control for SSTR2 staining. **Figure S5**. PET/CT images of ^64^Cu-SARTATE distribution in mice bearing intrahepatic neuroblastoma tumors 24 h post injection.

## Data Availability

The datasets generated during the current study are available from the corresponding author for collaborative purposes on reasonable request.
