# Supplementary material for: Detection and therapy of neuroblastoma minimal residual disease using [64/67Cu]Cu-SARTATE in a preclinical model of hepatic metastases
Source: EJNMMI Res. 2021 Feb 25;11:20. doi: 10.1186/s13550-021-00763-0 (PMC7907331; doi:10.1186/s13550-021-00763-0)
Supplement: Supplementary file 1 — Additional file 1. Supplementary data. Figure S1. Fluorescence-activated cell sorting (FACS) analysis of IMR32 neuroblastoma cell SSTR2 expression. Figure S2. Body weights of groups of mice following splenectomy and during therapy. Figure S3. Standard curve for sensitivity of the phosphor storage plates. Figure S4. Immunohistochemistry positive control for SSTR2 staining. Figure S5. PET/CT images of 64Cu-SARTATE distribution in mice bearing intrahepatic neuroblastoma tumors 24 h post injection. [file 13550_2021_763_MOESM1_ESM.doc]

**Detection and Therapy of Neuroblastoma Minimal Residual Disease**

**Using [^64/67^Cu]Cu-SARTATE in a Preclinical Model of Hepatic Metastases**

Jason L. J. Dearling^1^*, Ellen M. van Dam^2^, Matthew J. Harris^2^, and Alan B. Packard^1^

1. Division of Nuclear Medicine and Molecular Imaging, Department of Radiology, Boston Children’s Hospital, Boston MA 02115, USA; Harvard Medical School, Boston MA 02115, US;

2. Clarity Pharmaceuticals Ltd., 4 Cornwallis St., Sydney, New South Wales, 2015 Australia

**Additional file 1 – Supplementary data**

Figure S1. Fluorescence-activated cell sorting (FACS) analysis of IMR32 neuroblastoma cell SSTR2 expression.

Figure S2. Body weights of groups of mice following splenectomy and during therapy.

Figure S3. Standard curve for sensitivity of the phosphor storage plates.

Figure S4. Immunohistochemistry positive control for SSTR2 staining.

Figure S5. PET/CT images of ^64^Cu-SARTATE distribution in mice bearing intrahepatic neuroblastoma tumors 24 h post injection.

**
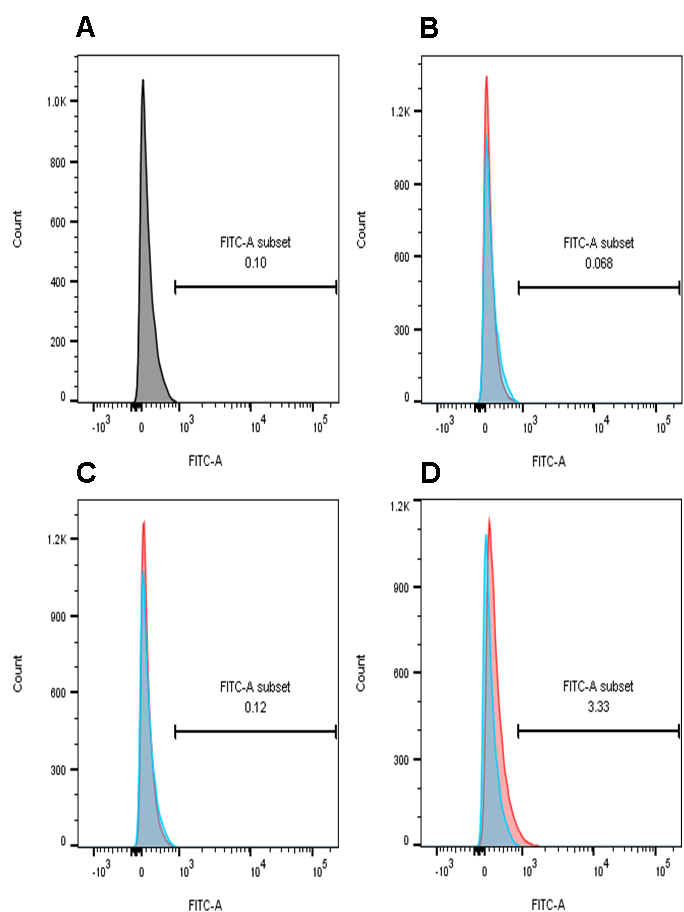
**

**Figure S1. Fluorescence-activated cell sorting (FACS) analysis of IMR32 neuroblastoma cell SSTR2 expression.** Data shown are: **A**) IMR32 cells alone; **B**) IMR32 cells stained with isotype-matched non-specific primary control; **C**) IMR32 cells incubated with anti-SSTR2 antibody at 2 µg/mL; **D**) IMR32 cells incubated with 1 µL of anti-SSTR2 stock antibody (undiluted), confirming expression of SSTR2 by the IMR32 cells.

**
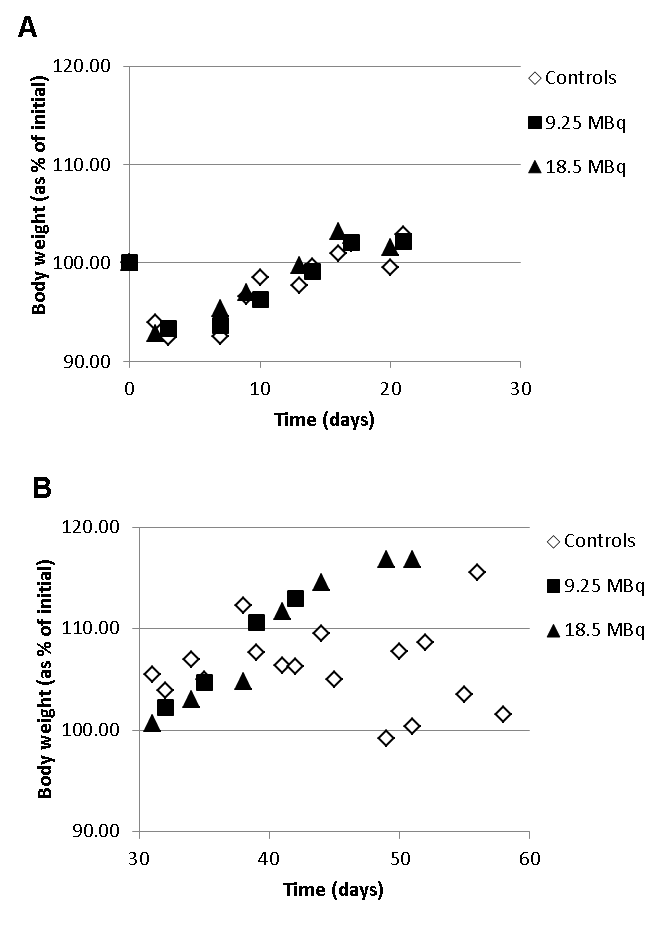
**

**Figure S2. Body weights of groups of mice following splenectomy and during therapy.** Figures S2**A** and S2**C** show the typical decrease of around 8% following splenectomy which the mice recover by about day 14. Figures S2**B** and S2**D** show the body weights for mice following injection with ^67^Cu-SARTATE. Figures S2A and B show the data for the first study (therapy started at 4 weeks following tumor cell inoculation) and Figures S2 C and D show the data for the second study (therapy started two weeks after tumor cell inoculation).


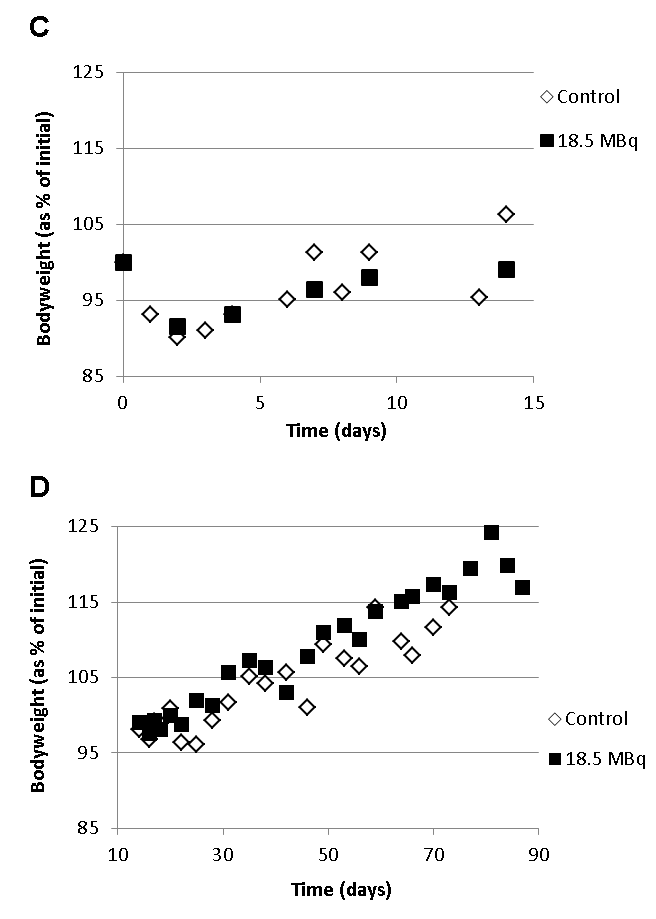


**
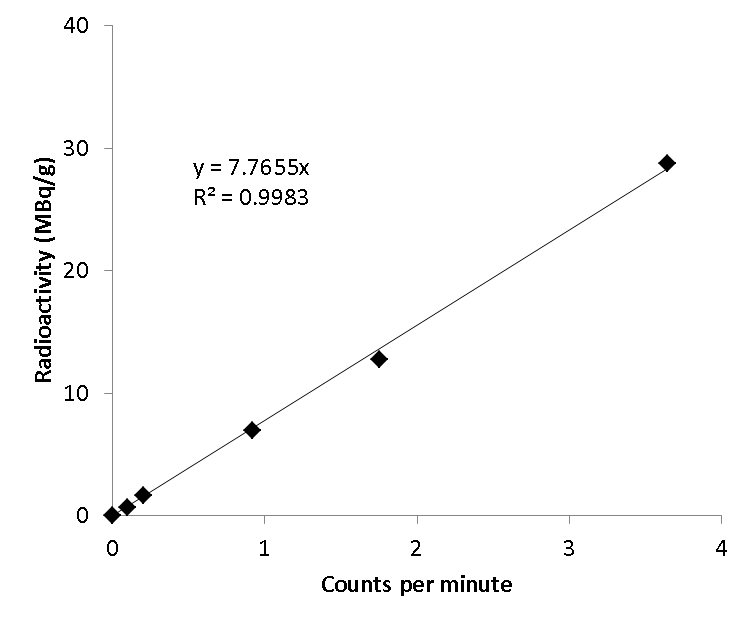
**

**Figure S3. Standard curve for sensitivity of the phosphor storage plates.** Standards of known activity and mass were frozen, 16 µm thick sections were exposed to phosphor storage plates, and resulting counts were converted to %ID/g by accounting for physical decay of the radionuclide and duration of exposure.

**
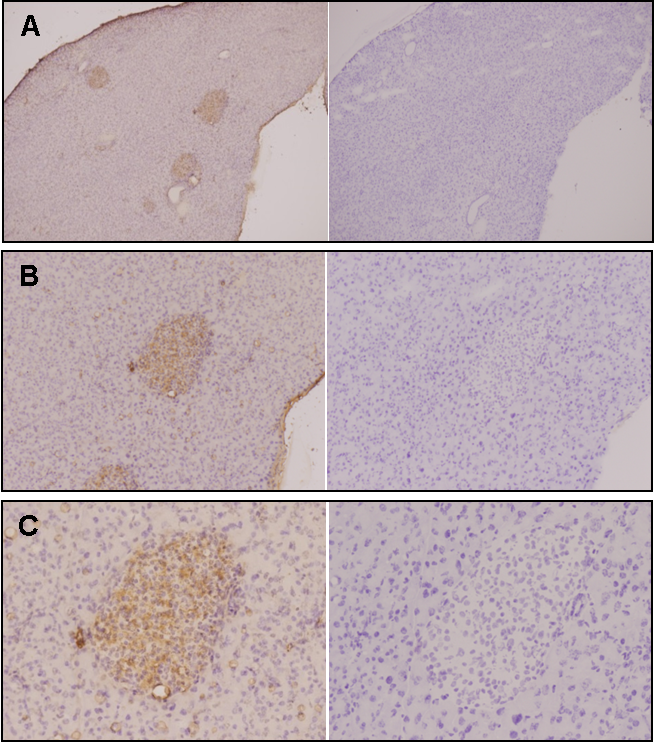
**

**Figure S4. Immunohistochemistry positive control for SSTR2 staining.** Images of frozen sections of mouse pancreas stained for SSTR2 as described for liver/tumor sections (*left column*), and corresponding primary omission negative controls carried out on contiguous sections (*right column*). Photographs were taken at 40x, 100x and 200x magnification (A, B and C, respectively).


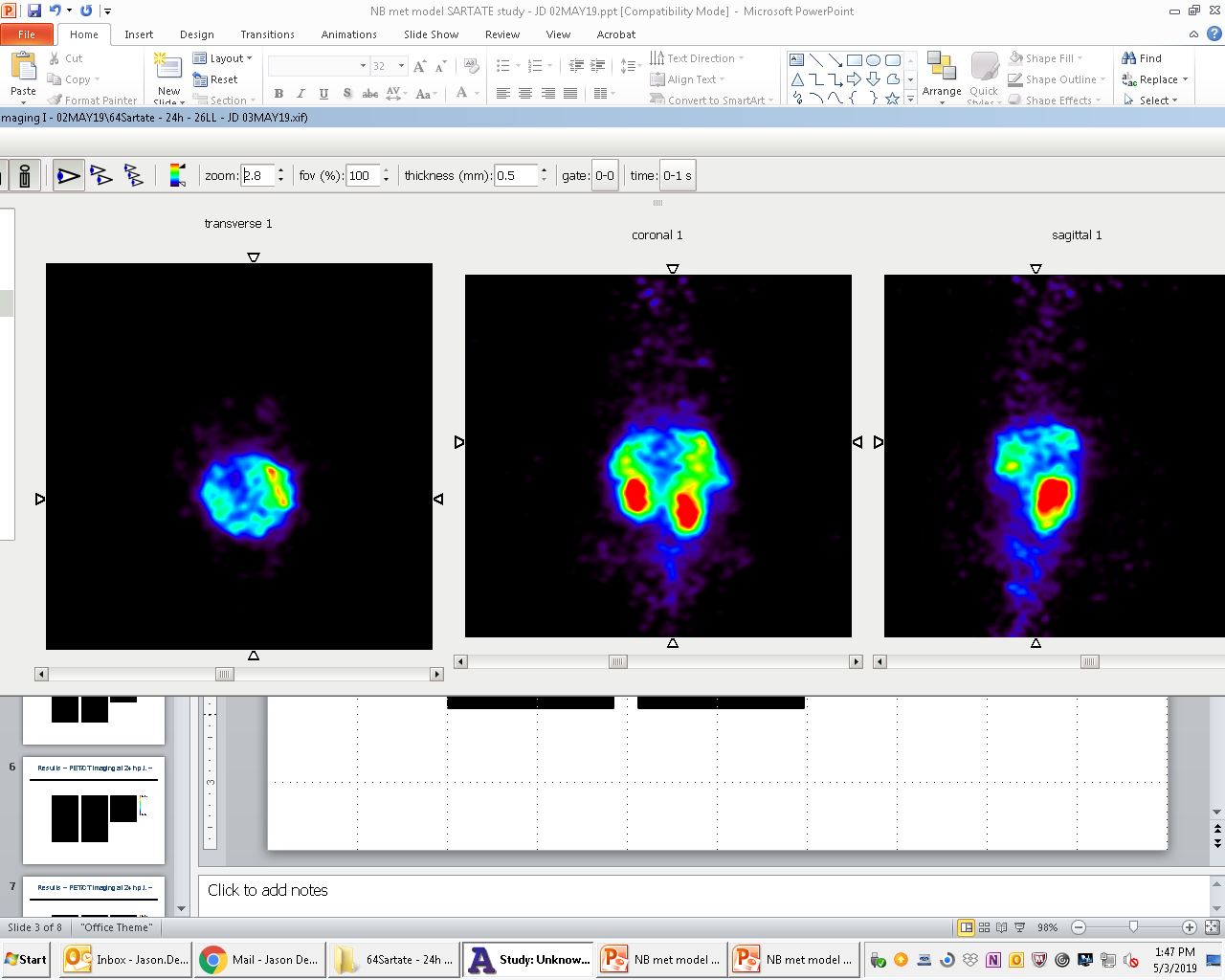

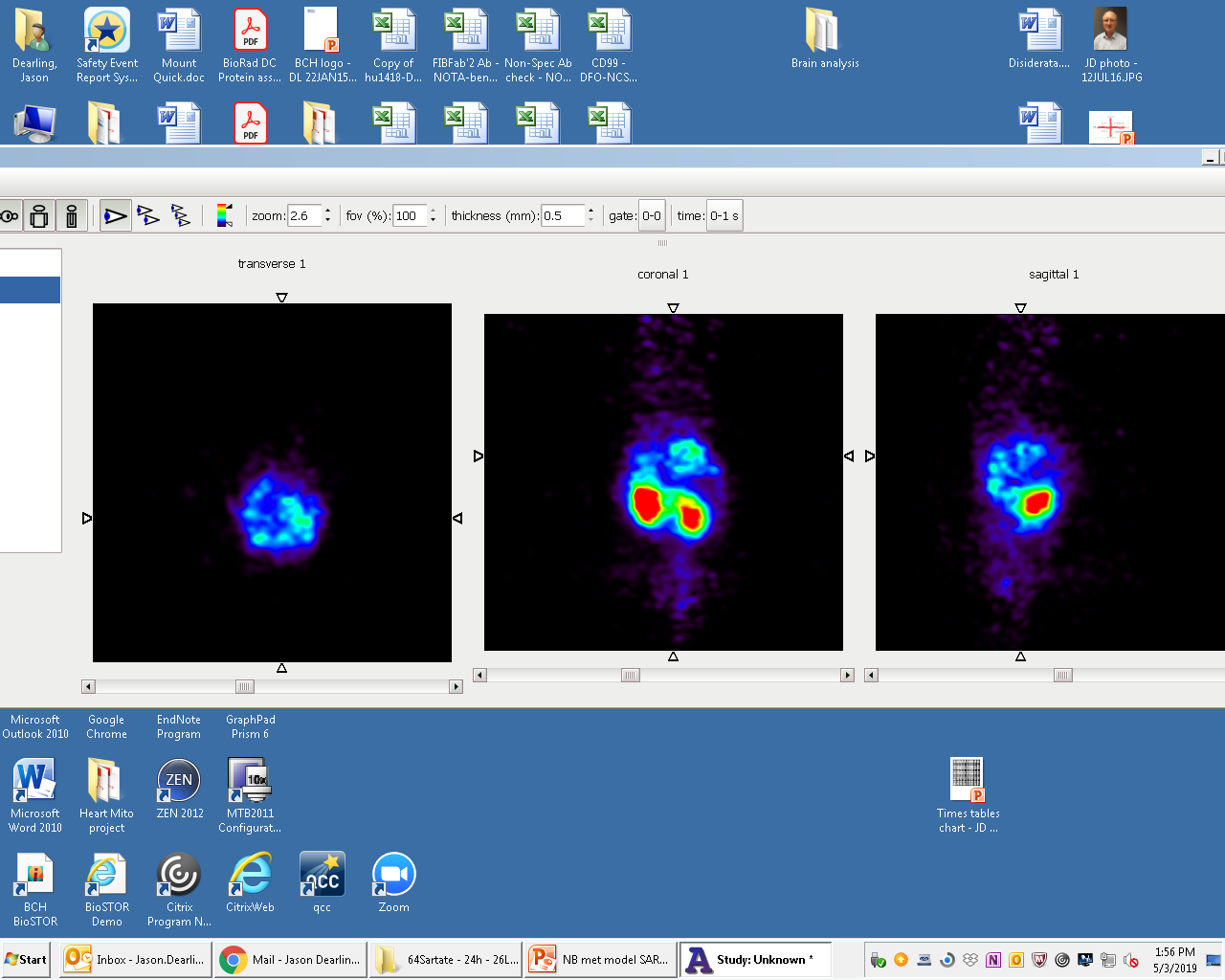

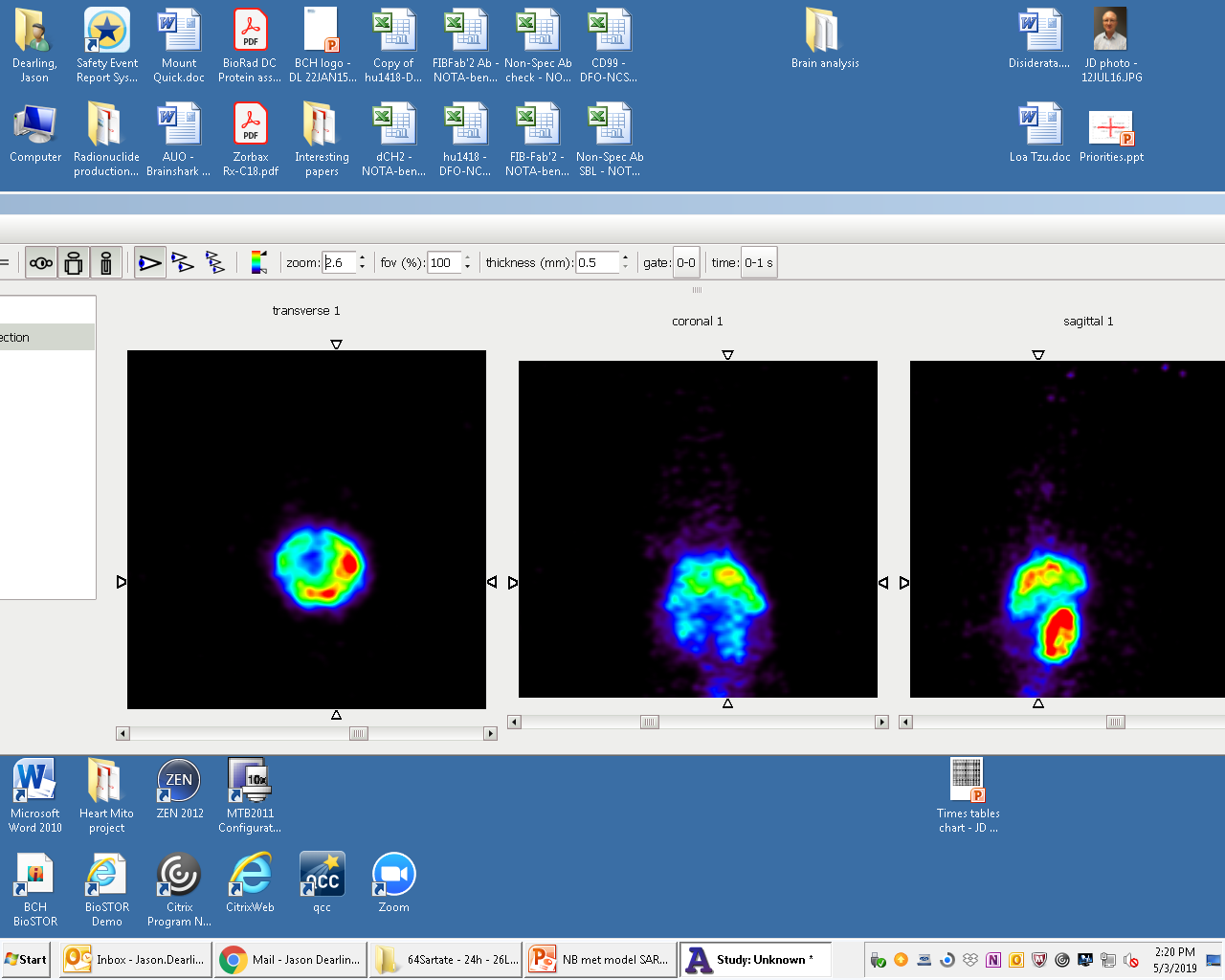

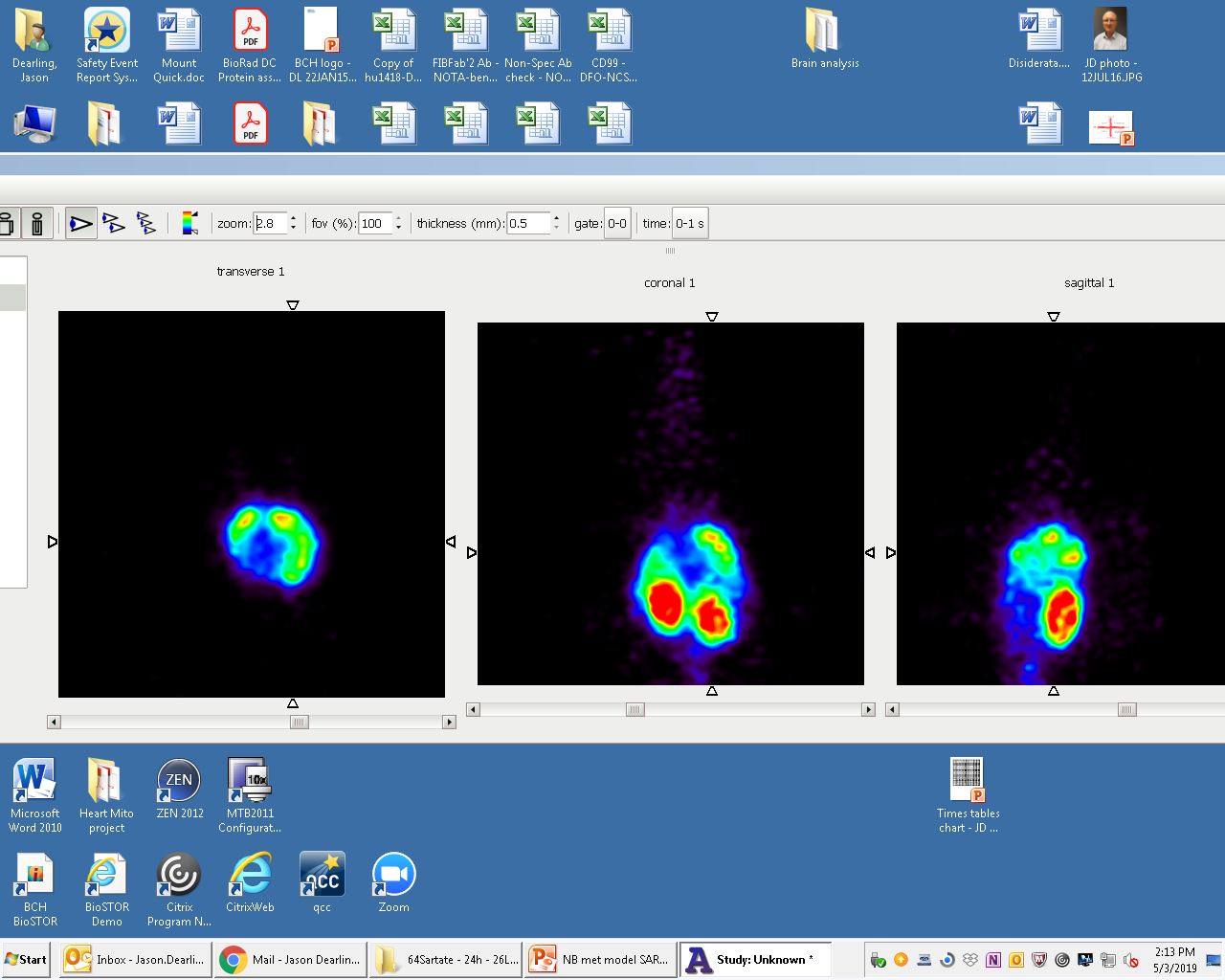

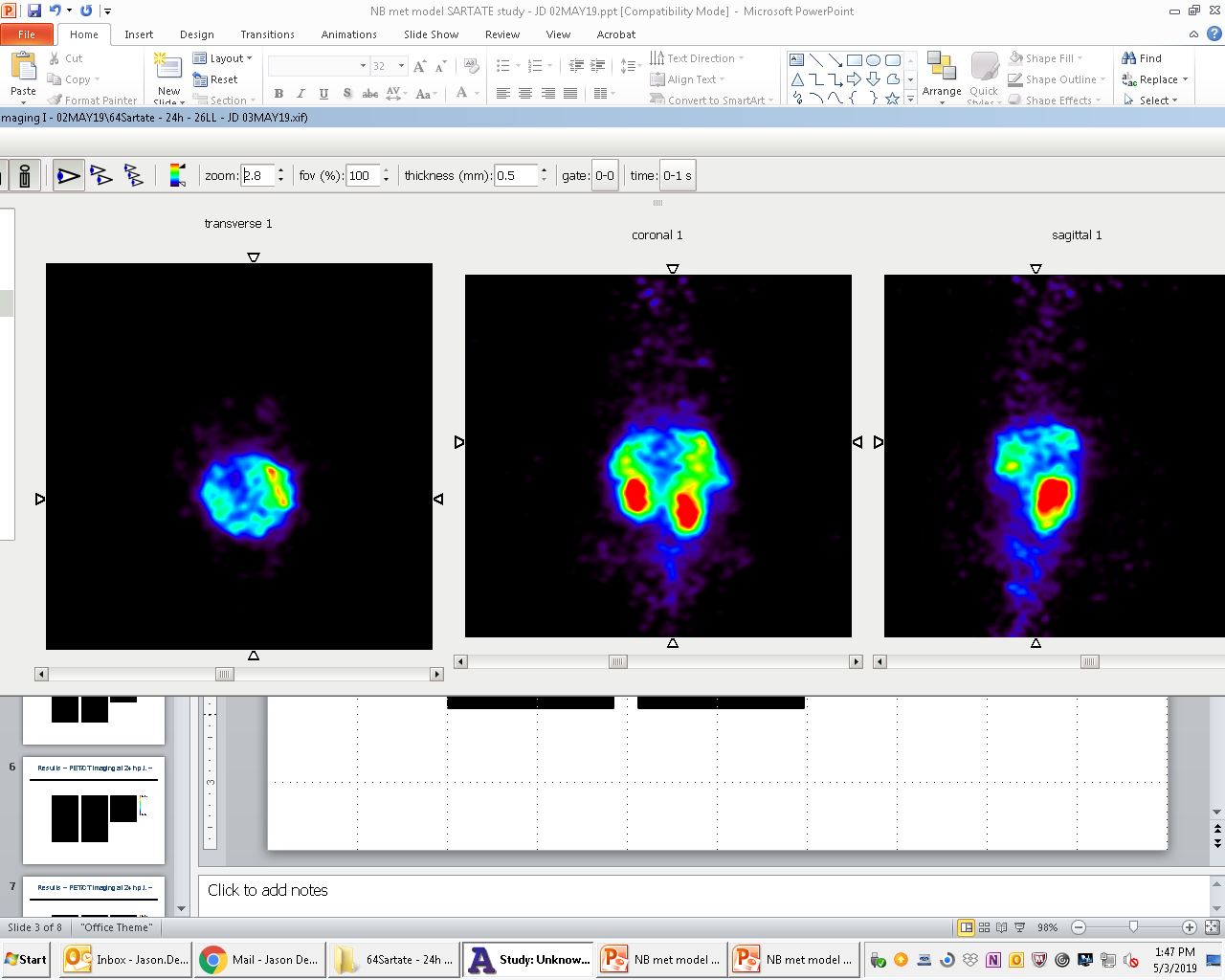

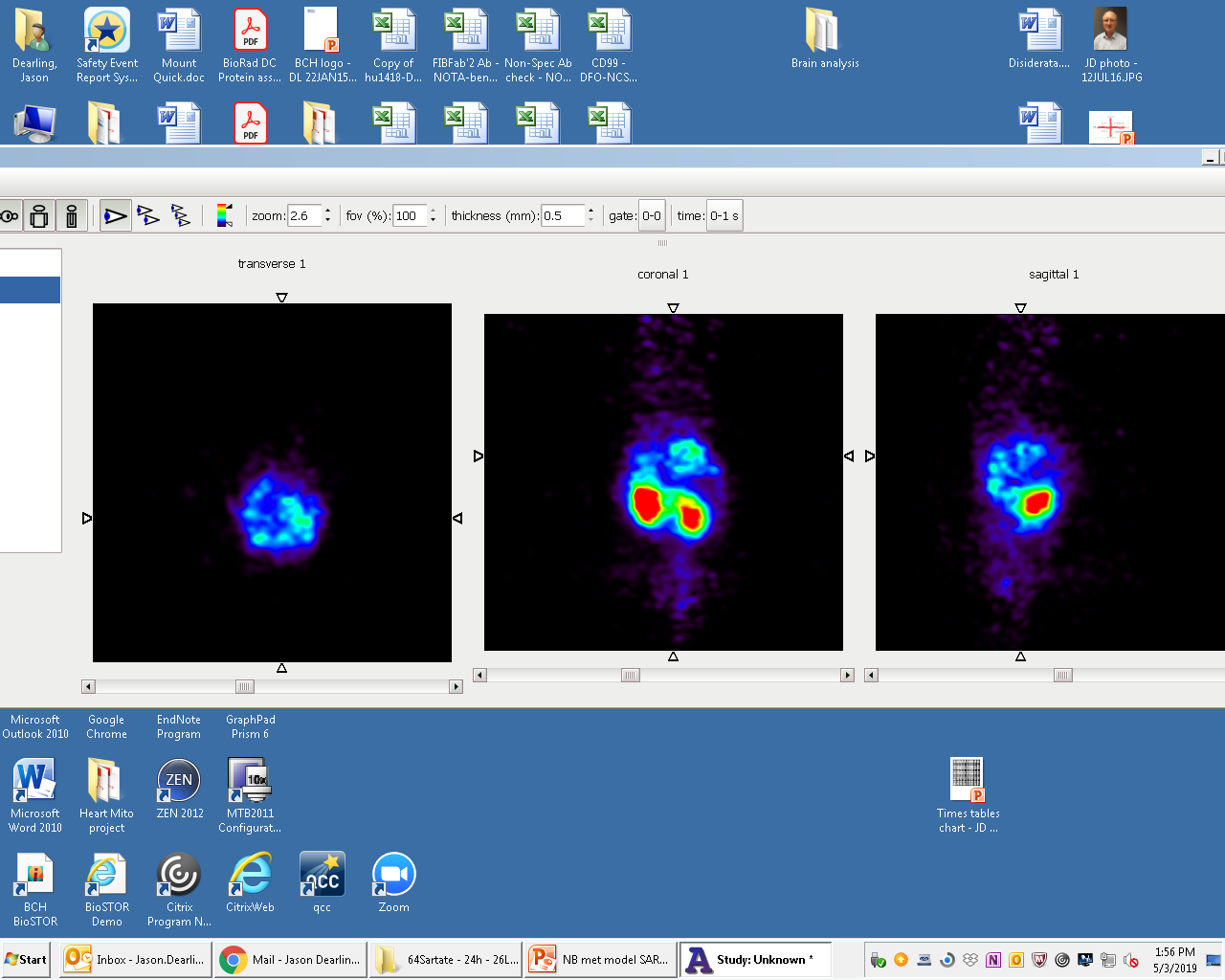

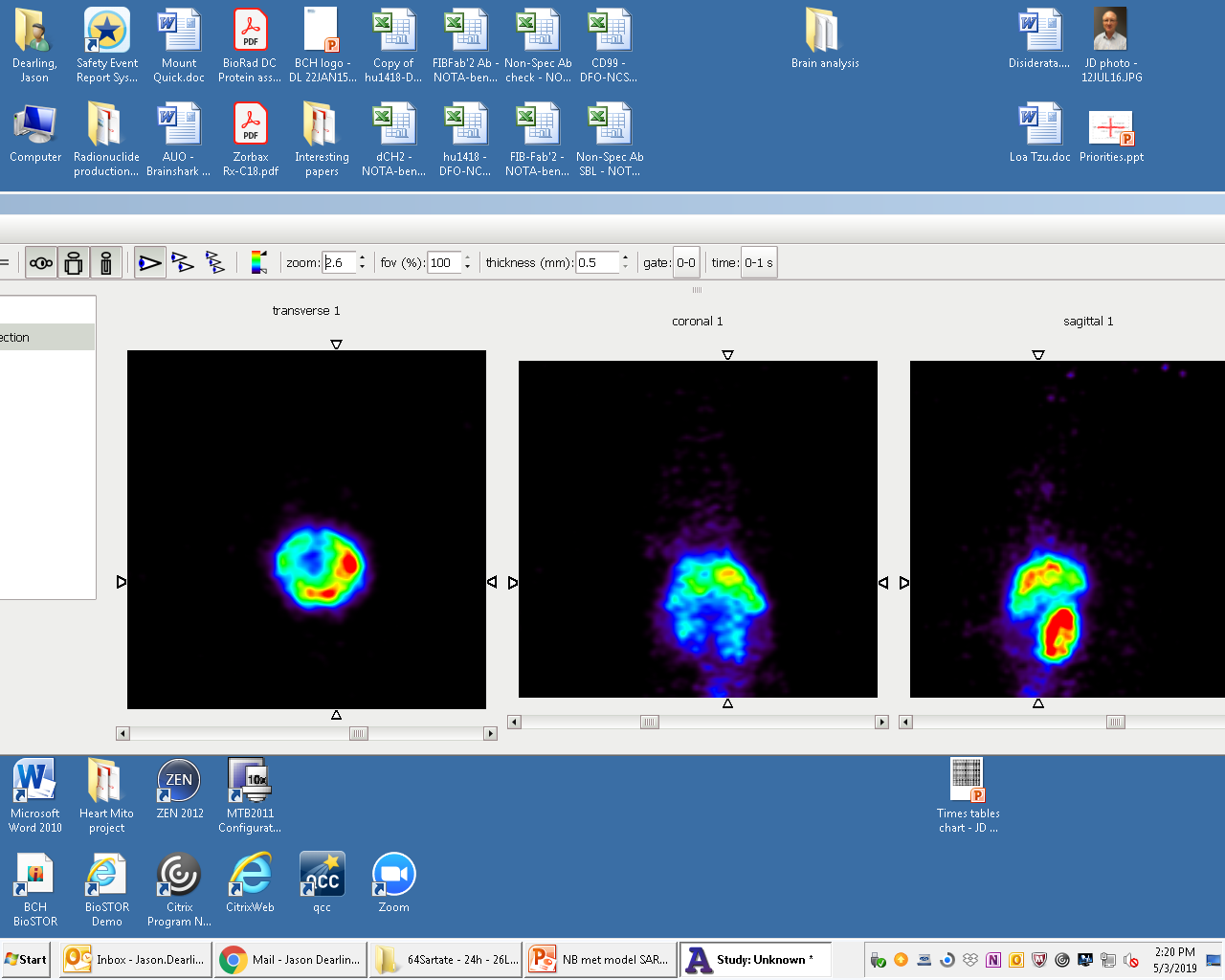

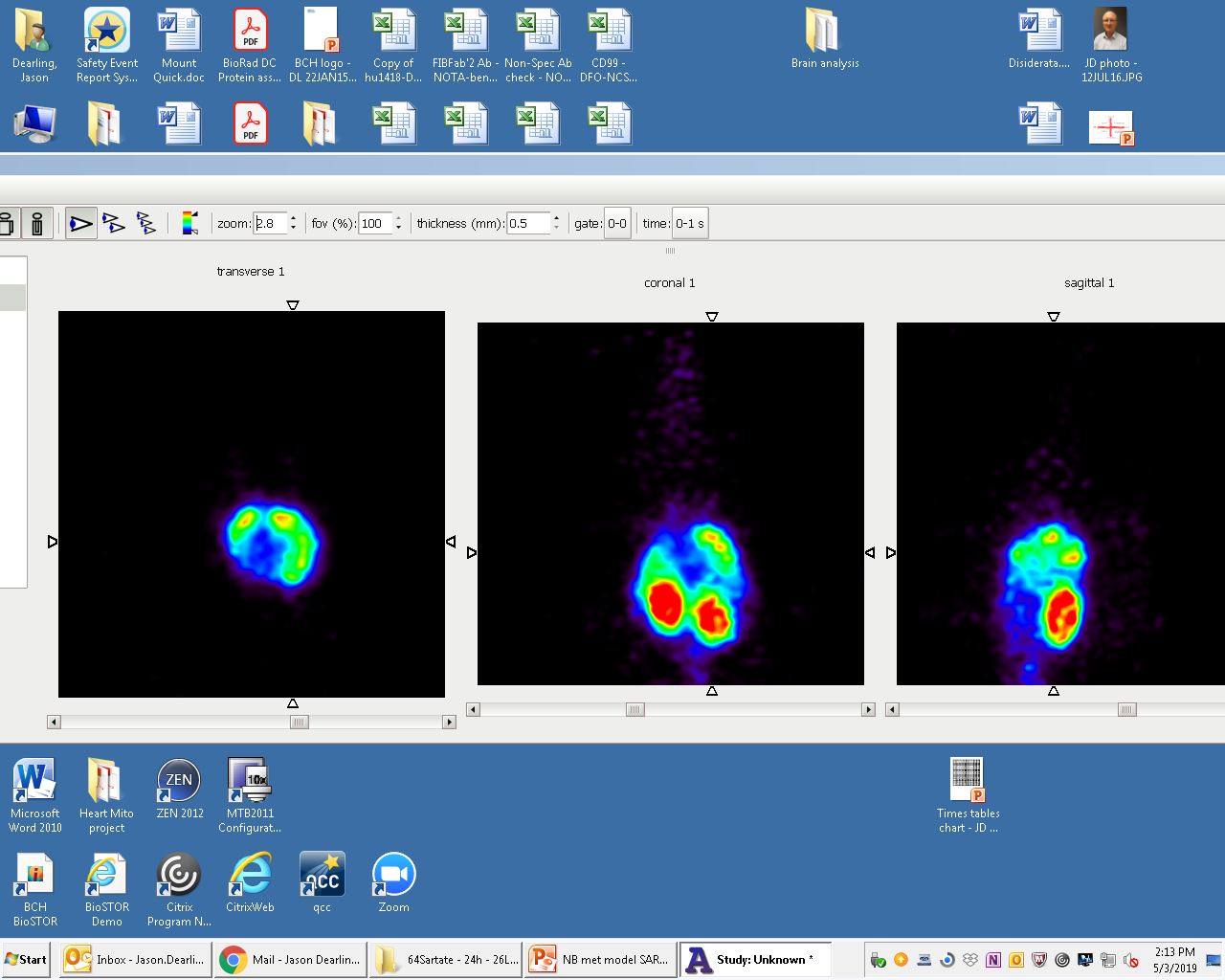

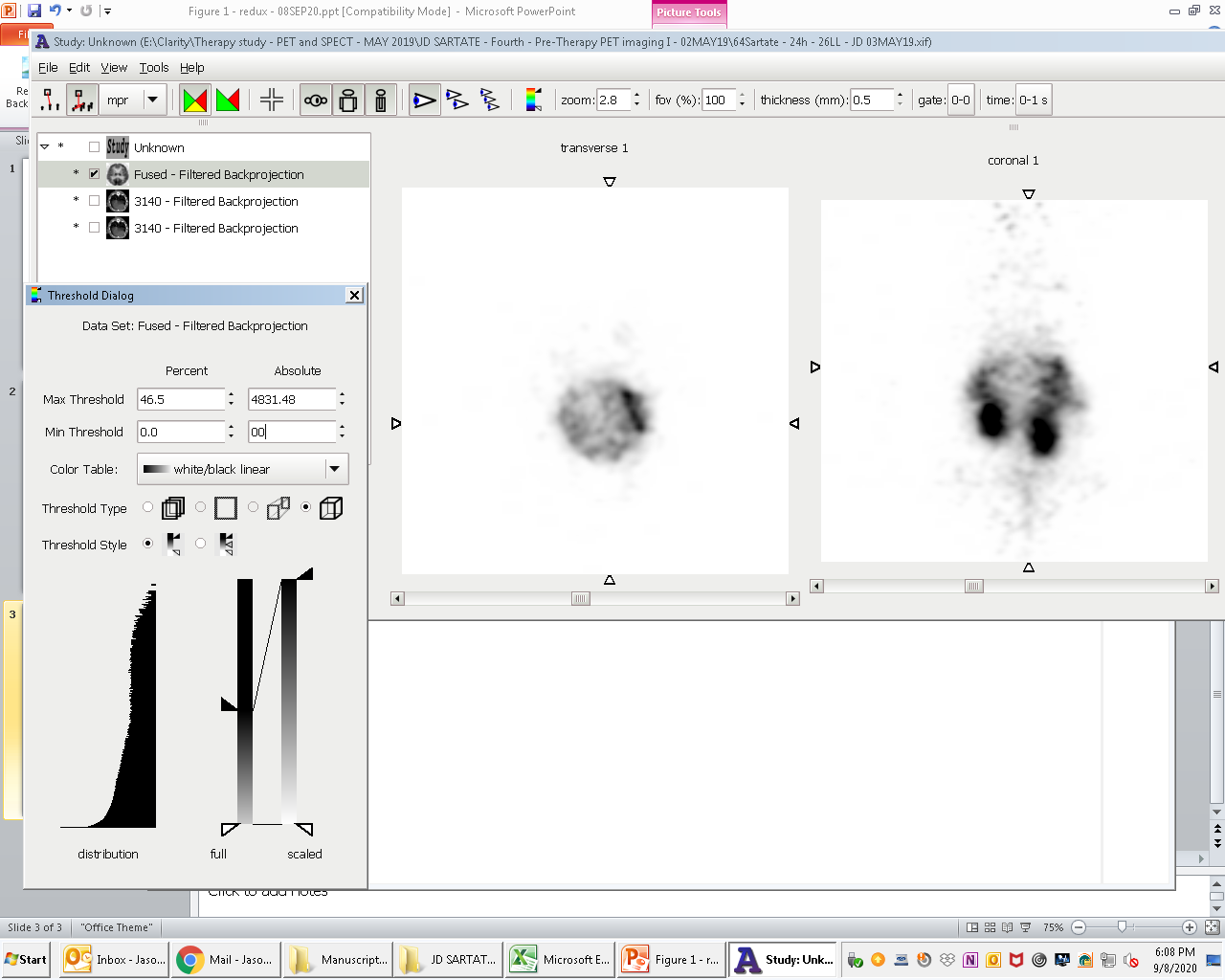

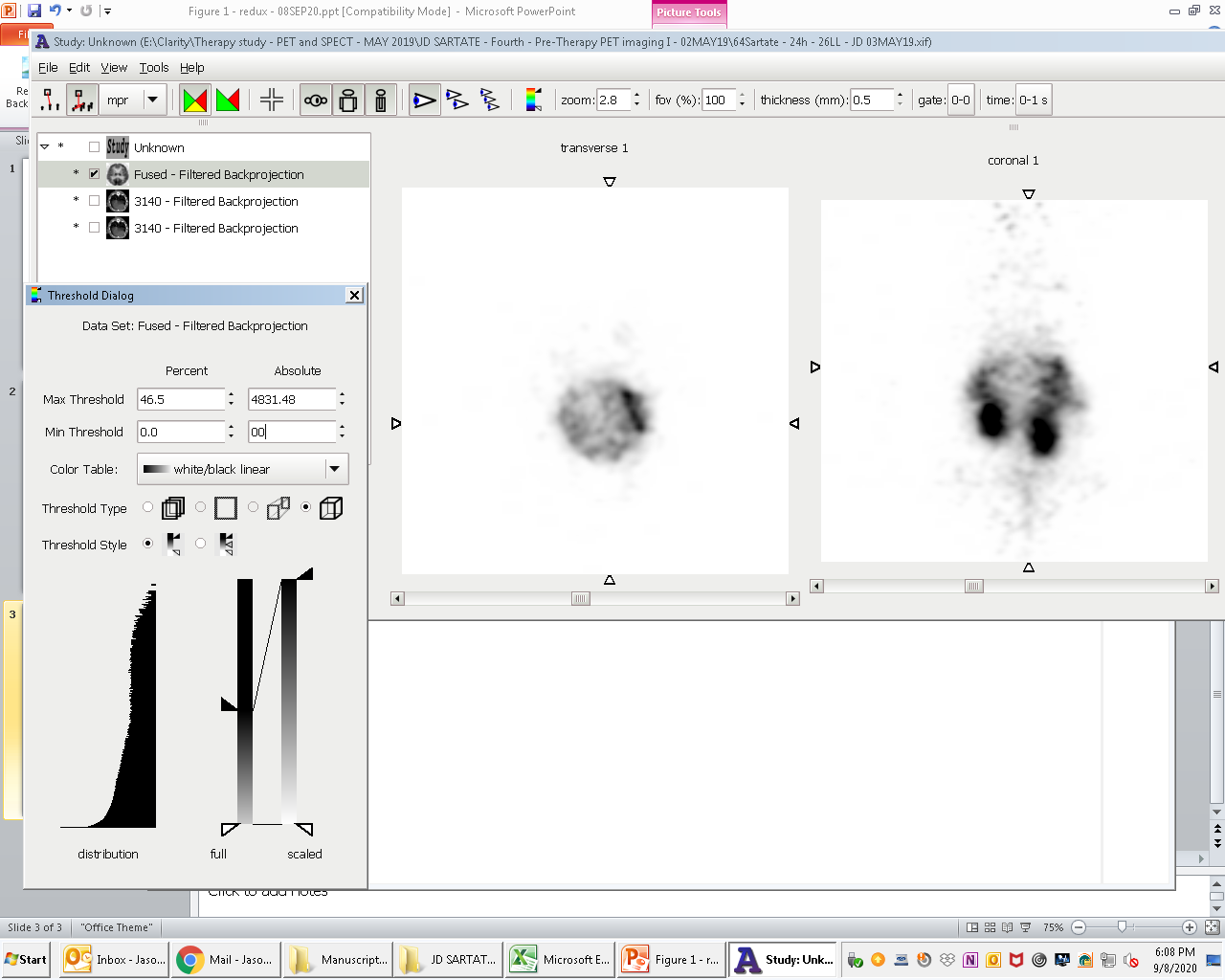

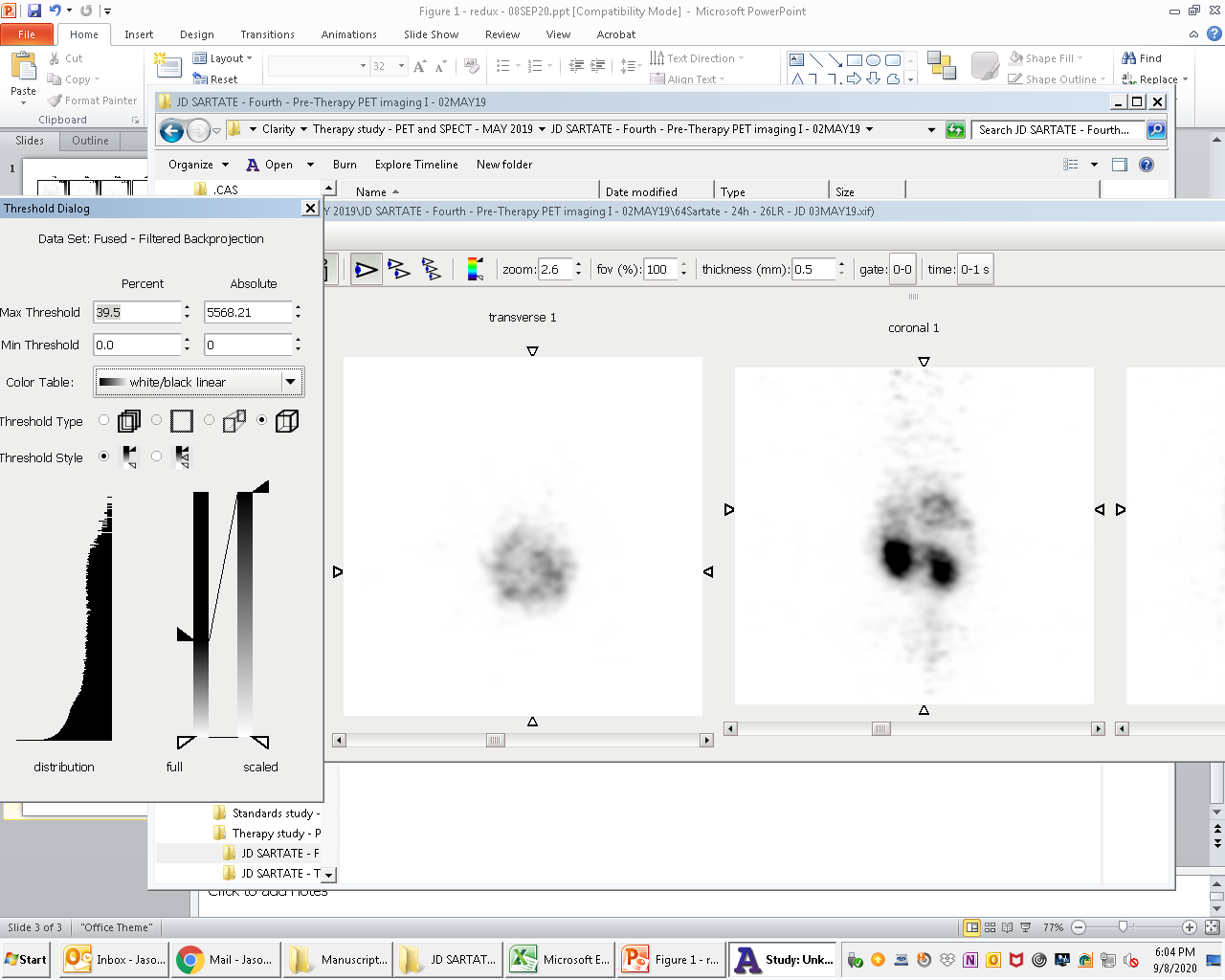

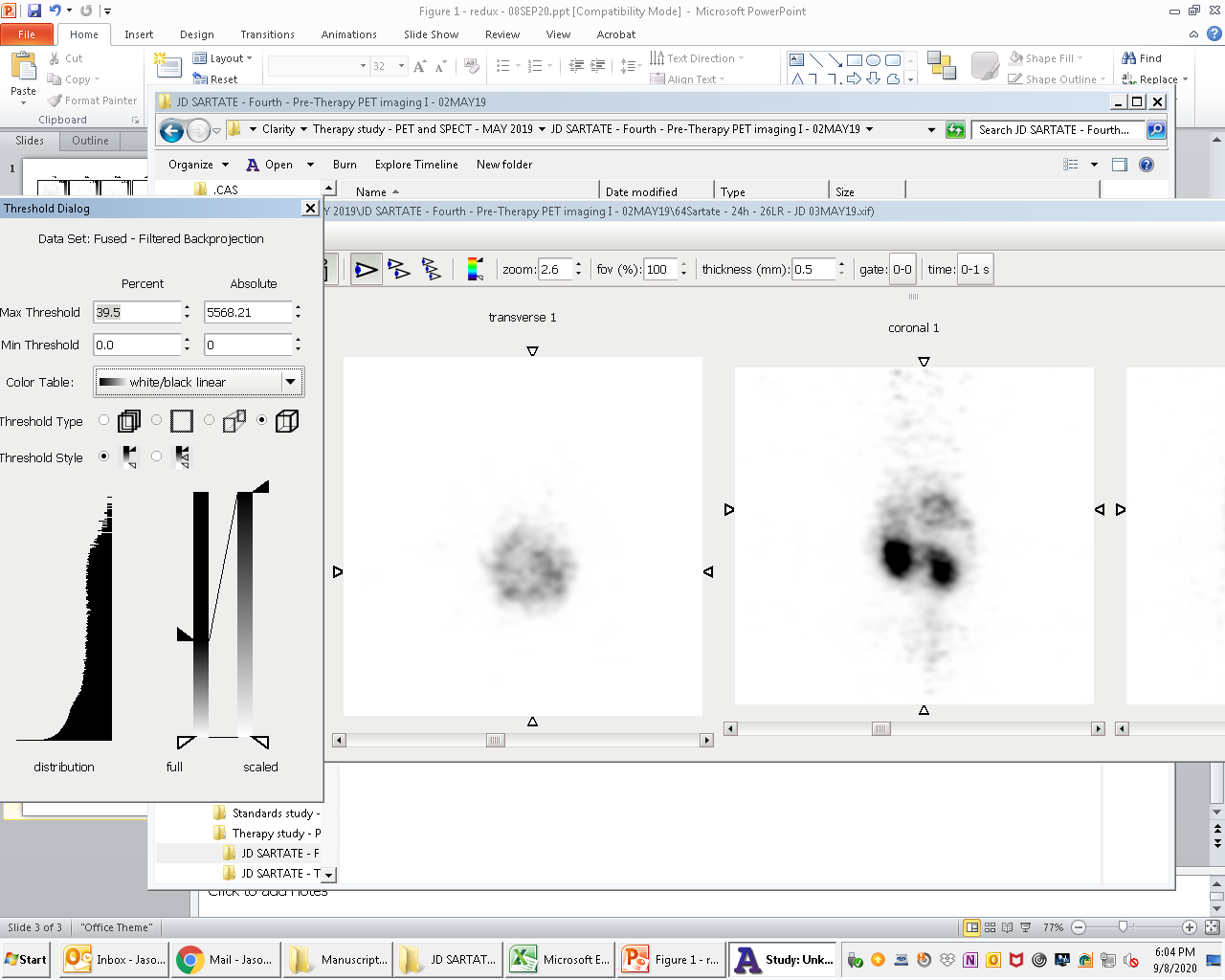

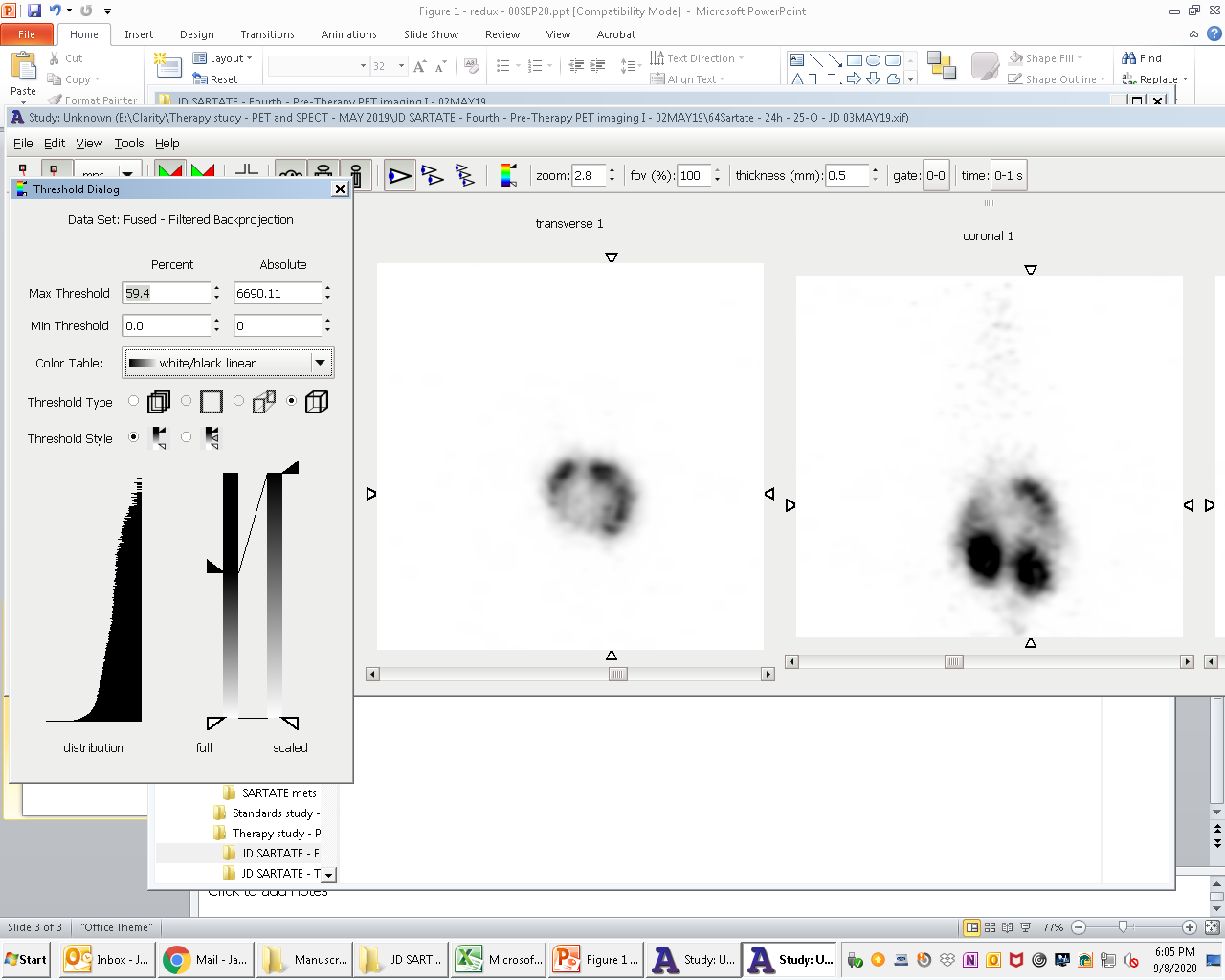

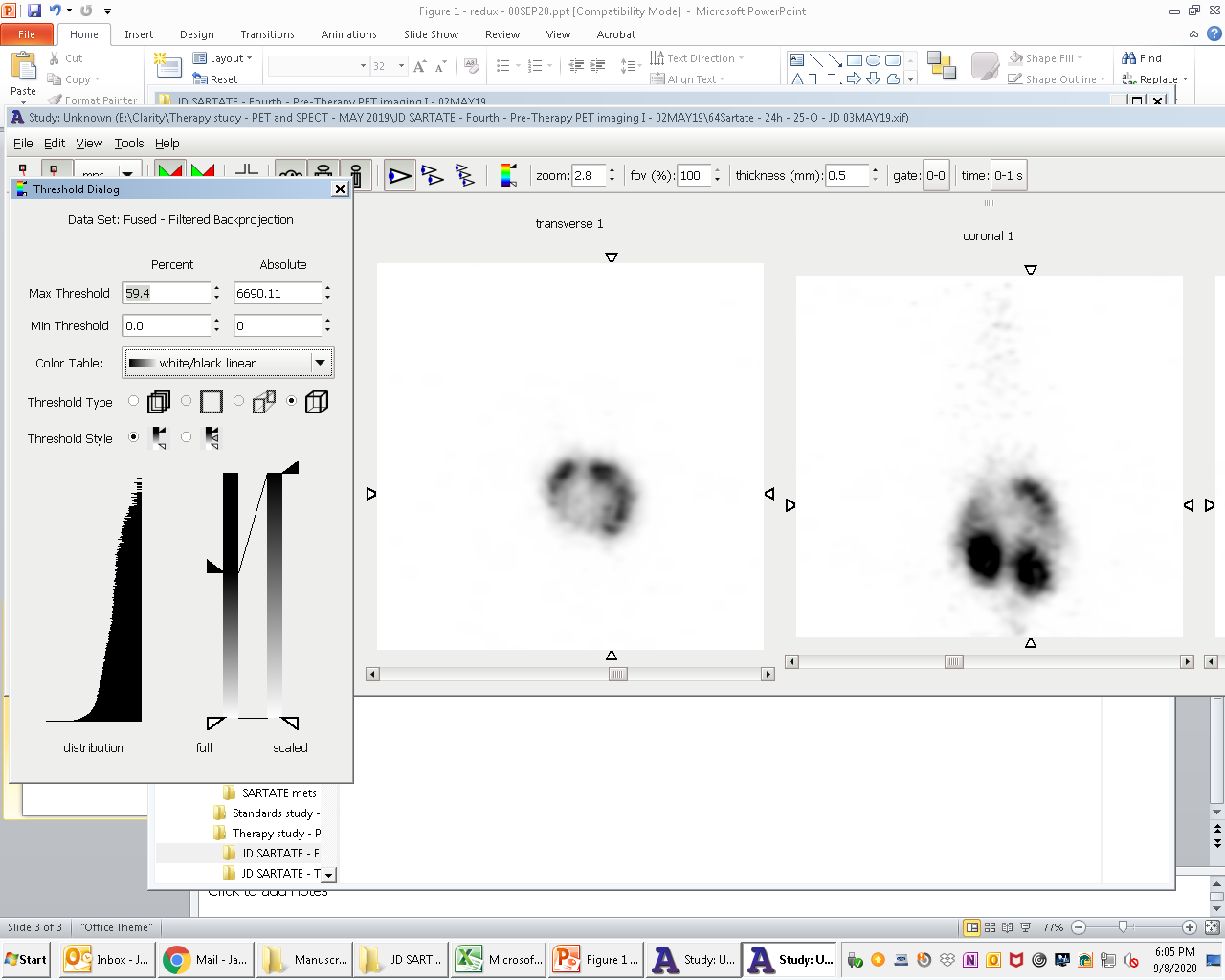

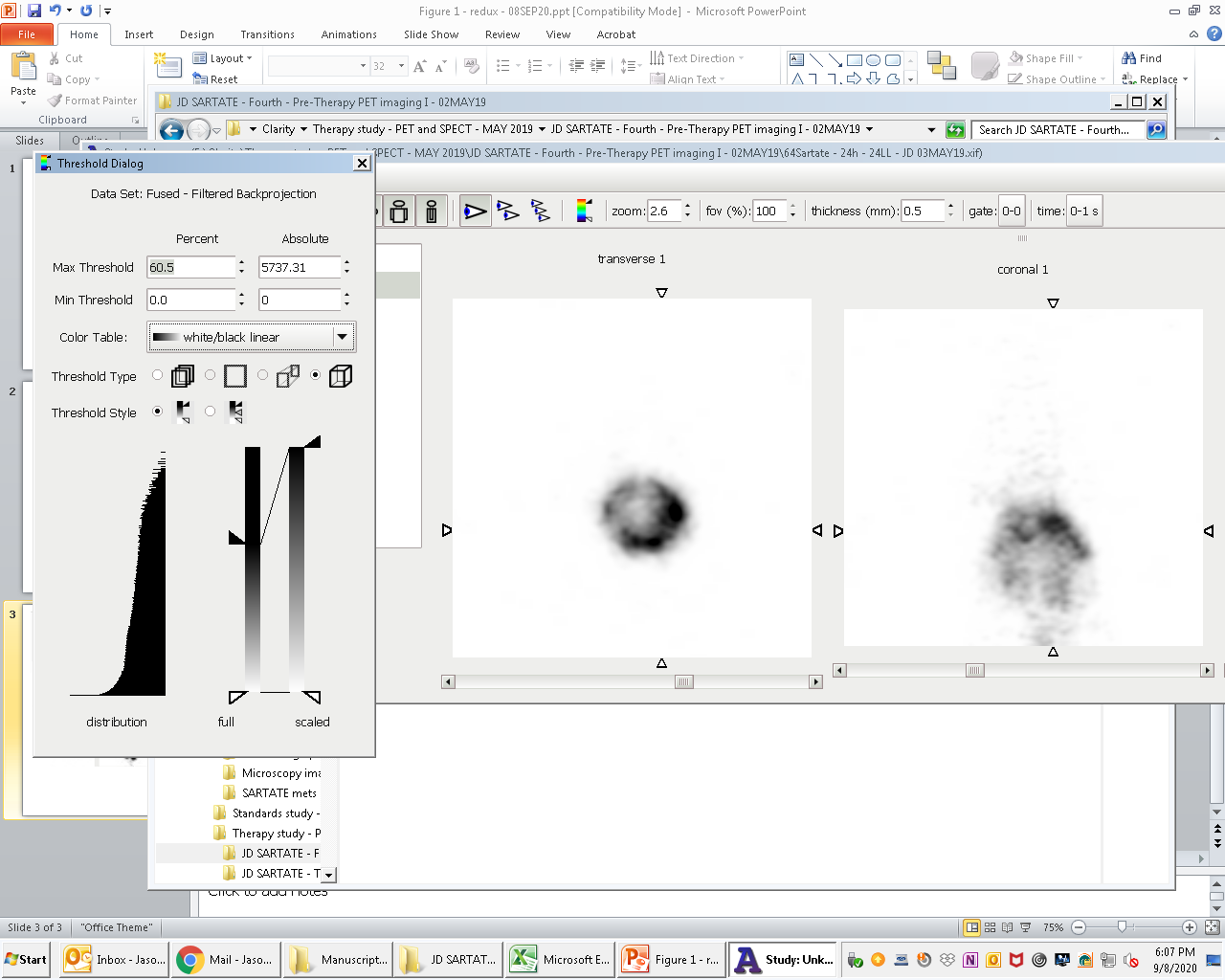

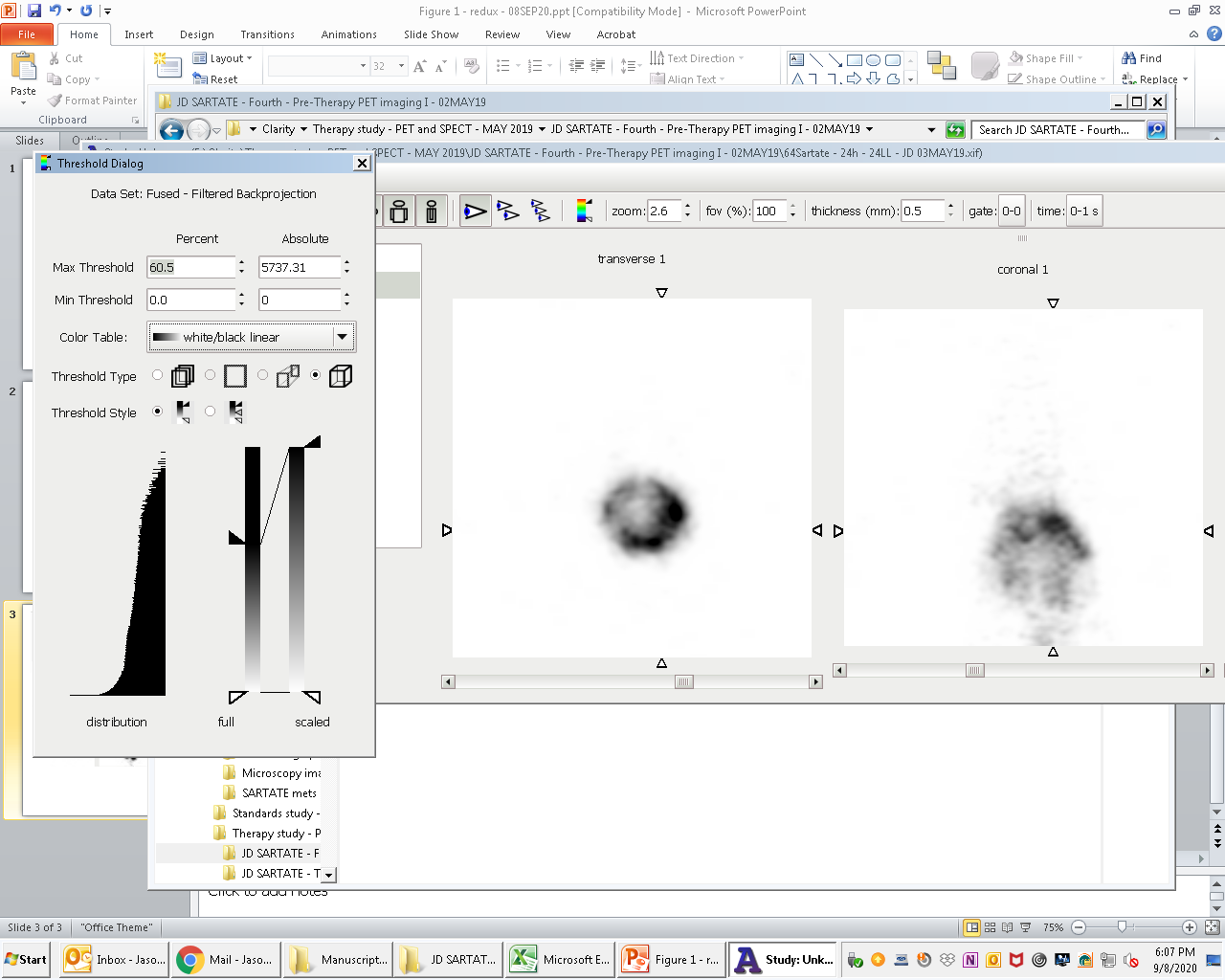

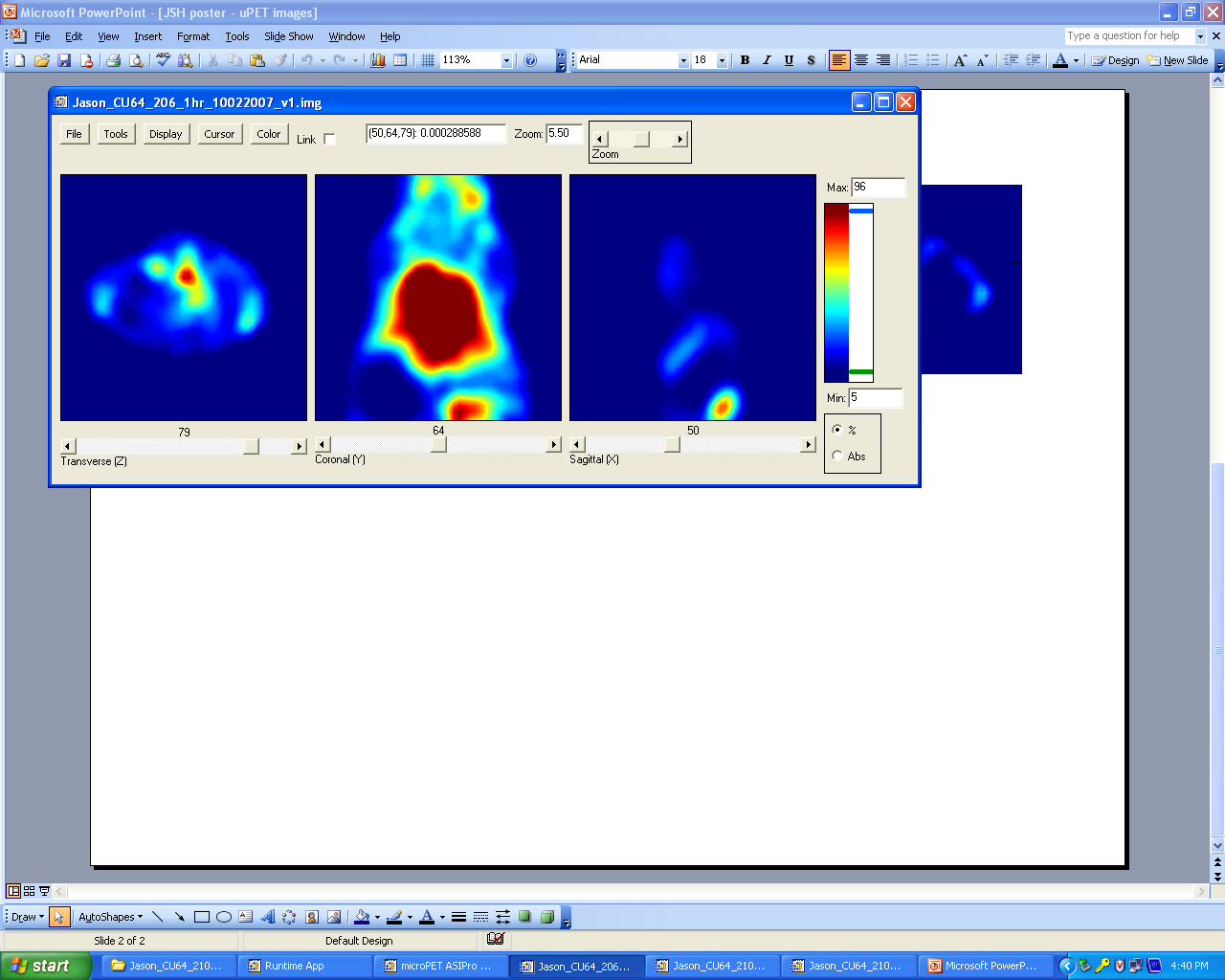


**Head**

**Tail**

**1h**

**5h**

**24h**

**48h**


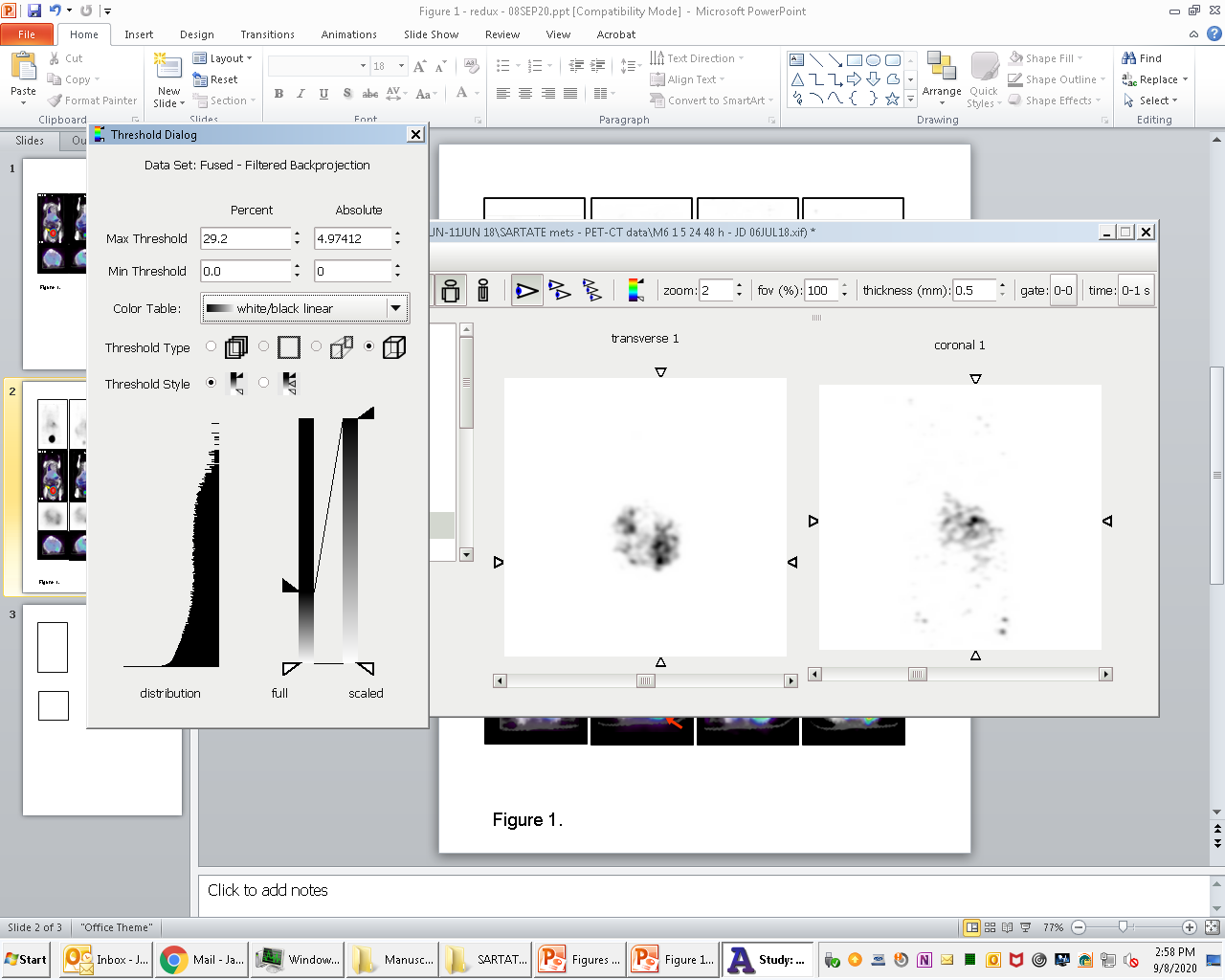


**0**

**1.16**


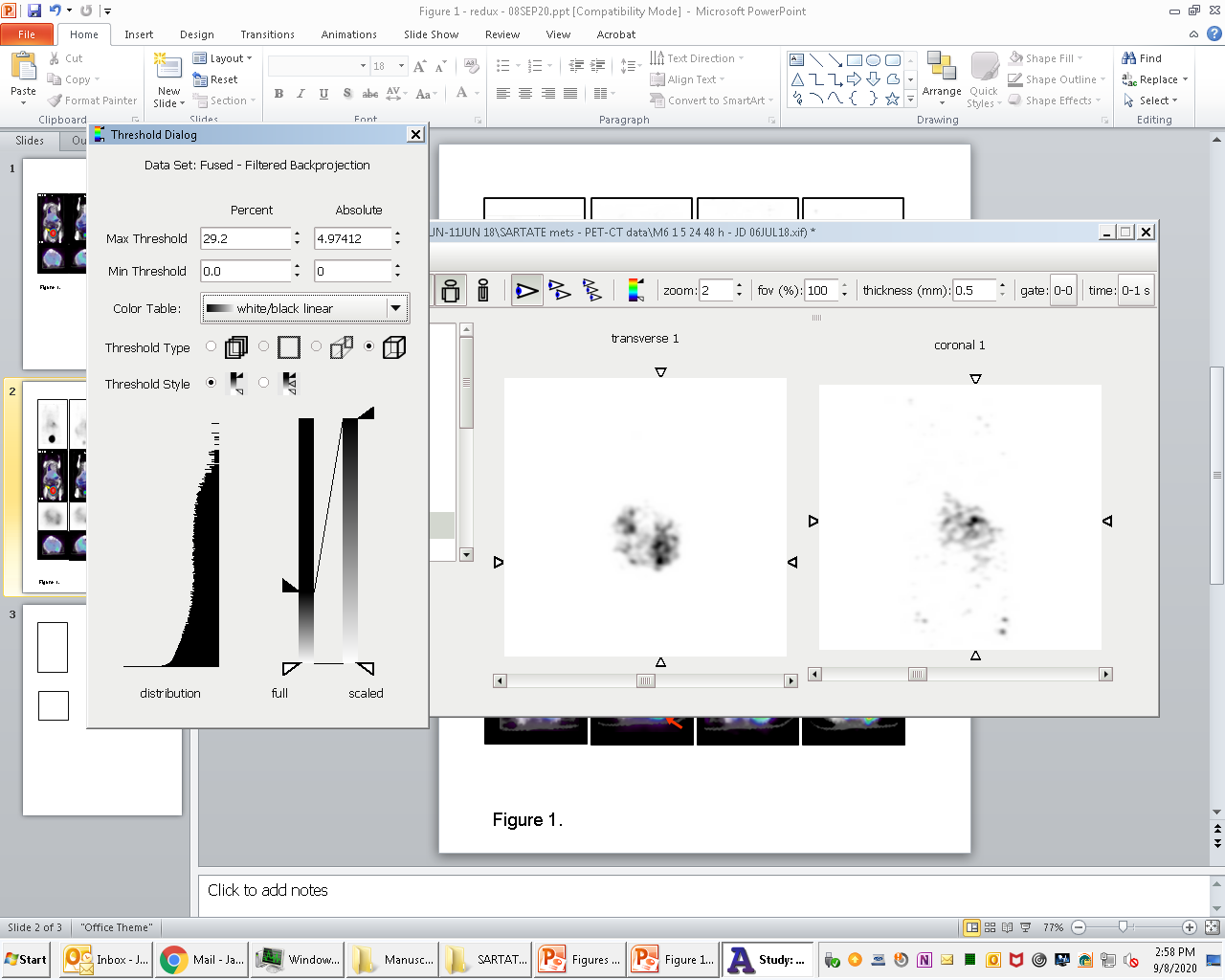


**0**

**1.59**


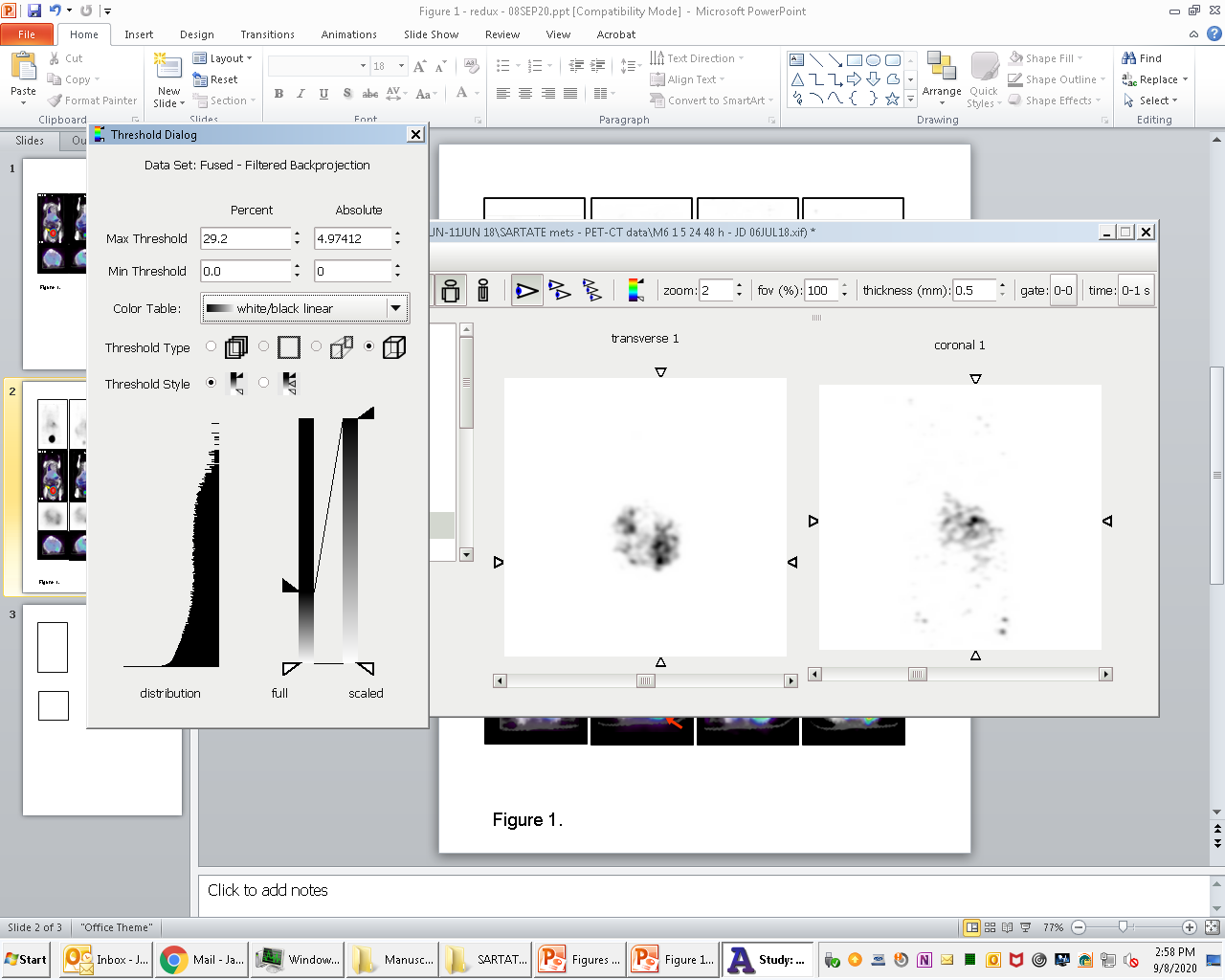


**0**

**1.85**


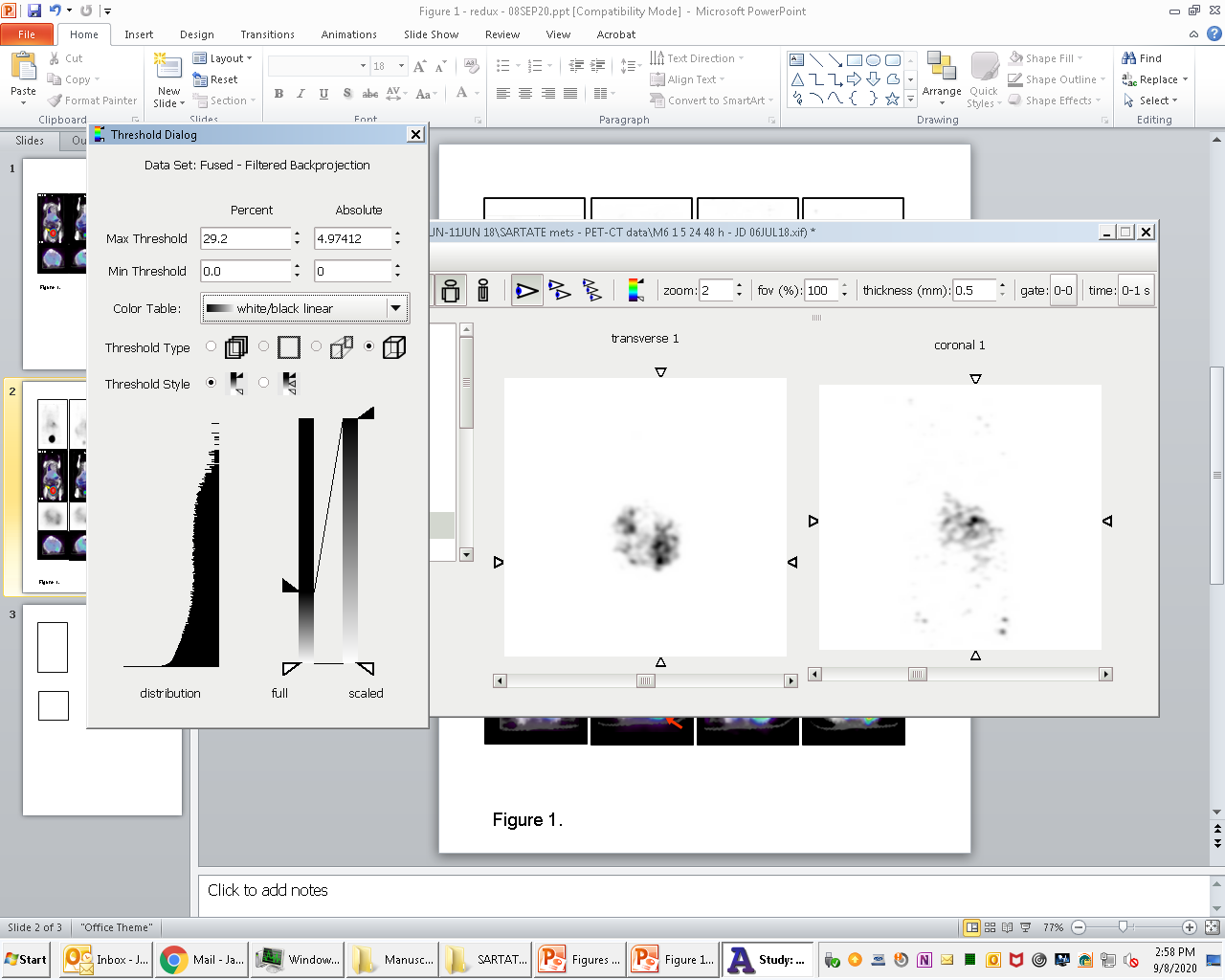


**0**

**1.42**

**Figure S5. PET/CT images of ^64^Cu-SARTATE distribution in mice bearing intrahepatic neuroblastoma tumors 24 h post injection.** After 3 weeks of tumor growth, mice that were going to be treated with ^67^Cu-SARTATE at 4 weeks were imaged using ^64^Cu-SARTATE. Two mice from each group were imaged and examples are shown. As with the biodistribution study, kidney uptake was high (white arrow). High and heterogeneous uptake in the liver (*e.g.* yellow arrows) was interpreted as confirmation of tumor growth. *Top and third row coronal images, second and bottom row transaxial images through the liver/tumor. PET data scale bar in gray scale calibrated to %ID/g.*
